# Biological control of nosemosis in *Apis mellifera* L. with *Acacia nilotica* extract

**DOI:** 10.1038/s41598-024-78874-6

**Published:** 2024-11-16

**Authors:** Ashraf S. A. El-Sayed, Nahla A. M. Fathy, Mai Labib, Ashraf F. El-Baz, Aly A. El-Sheikh, Ahmed H. Moustafa

**Affiliations:** 1 Enzymology and Fungal Biotechnology Lab (EFBL), Botany and Microbiology Department, Faculty of Science, Zagazig University, Zagazig, 44519 Egypt; 2 Plant Protection Research Institute, Agricultural Research Center, Dokki, Giza, Egypt; 3 Agriculture Genetic Engineering Research Institute (AGERI), Agricultural Research Center, Giza, 12619 Egypt; 4 Genetic Engineering and Biotechnology Research Institute, University of Sadat City, Sadat City, 22857/79 Egypt; 5 Chemistry Department, Faculty of Science, Zagazig University, Zagazig, 44519 Egypt

**Keywords:** *Nosema* spp, Honeybees, *Apis mellifera*, Nosemosis, *Acacia nilotica*, *Catharanthus roseus*, GC–MS analysis, Microbiology, Zoology, Entomology

## Abstract

Nosemosis is one of the most devastating diseases of *Apis mellifera* (Honey bees) caused by the single-celled spore-forming fungi *Nosema apis*, *N. ceranae* and *N. neumanii,* causing a severe loss on the colony vitality and productivity. Fumagillin, a MetAP2 inhibitor, was a certified treatment for controlling nosemosis, nevertheless, due to its deleterious effects on honey bees and humans, it is prohibited. So, searching for novel biological agents with affordable selectivity to target *Nosema* species infecting *Apis mellifera*, with nil toxicity to bees and humans is the main objective of this study. *Nosema* species were isolated from naturally infected honey bees. The methanolic extracts of *Acacia nilotica*, *Elaeis guineensis*, and *Catharanthus roseus* were tested to selectively control the growth of *Nosema* spp of honeybees. The spores of *Nosema* species were molecularly and morphologically identified. Among the tested plant extracts, the methanolic extracts (0.1%) of *A. nilotica* had the most activity towards *Nosema* spp causing about 37.8 and 32.5% reduction in the spores’ load at 5- and 9-days post-infection, respectively, compared to the untreated control. At 0.1%, the *A. nilotica* methanolic extract exhibited the highest inhibitory effect for *Nosema* spores, without any obvious bee mortality. *Catharanthus roseus* displayed a reduction of spores by 27.02%, with bee mortality rate of 27.02%. At 1% for 5 dpi, the *A. nilotica* extracts led to 18.18% bee mortality, while the *C. roseus* extracts resulted in 100% mortality, as revealed from the toxicity and quantification bioassays. So, the extracts of *A. nilotica* and *C. roseus* had a significant effect in controlling the *N. apis* and *N. ceranae* titer compared to the infected untreated control at both time points. The titer of *N. apis* and *N. ceranae* was noticeably decreased by more than 80% and 90%, in response to *A. nilotica*, compared to the control. From the metabolic profiling by GC–MS analysis, the most frequent active compounds of *A. nilotica* were 2,4,6-trihy-droxybenzoic acid, 1,2-dihydroxybenzene, myristic acid, and linoleic acid. These compounds were analyzed in silico to assess their binding affinity to the ATP binding protein, methionine aminopeptidase and polar tube protein of *Nosema* species as target enzymes. The compound 2,4,6-trihydroxybenzoic acid had the lowest energy to bind with ATP binding protein, methionine aminopeptidase and polar tube protein of *Nosema*, followed by 1,2-dihydroxybenzene and myristic acid, compared to fumagilin. So, from the experimental and molecular docking analysis, the extracts of *A. nilotica* had the highest activity to attack the cellular growth machinery of *Nosema* species without an obvious effect to the honeybees, ensuring their prospective promising application.

## Introduction

Honeybees (*Apis mellifera*) are one of the most economically important insects for human by providing honey and beeswax for nutrition and medication. However, the honeybees are vulnerable to various biotic stressors like bacteria, viruses, and fungi, causing a significant colony loss and Colony Collapse Disorder (CCD). CCD is characterized by high local colony mortality, rapid depopulation, and the lack of known symptoms^1^. Nosemosis is an intestinal disease of the adult honey bee, that can caused by indigenous pathogen *Nosema apis*, *N. ceranae* and *N. neumanii*^2–5^*,* as fungal microsporidia^6^. Furthermore, a third type, *N. neumanii*, was recently discovered in Uganda and found to be phylogenetically related to *N. apis,* without any specific clinical symptoms^7,8^ The genus *Nosema* was recently re-identified as *Varirmorpha* according to^9^. The infection occurs through oral ingestion of spores, that germinate to a single-celled vegetative stage of the parasite which colonizes and reproduces within the mid-gut cells, causing an intestinal wall destruction ^10,11^. Microsporidia are a diverse group of intracellular parasites that infect both invertebrates and vertebrates ^7,12^^.^ Nosemosis disease could occur in adult honeybees, including workers, drones and queens ^3,13,14^. *Nosema* infection primarily deteriorates the gut epithelial cells of honeybees, leading to compromised nutrient absorption, decreased longevity, and impaired foraging behavior. Infected bees exhibit a reduced ability to metabolize food, resulting in weakened immune responses, energetic stress, and increased susceptibility to other stressors such as pesticides and pathogens. Furthermore, *Nosema*-infected colonies may experience reduced brood rearing and colony productivity^15–17^. In contrast, honeybees have developed various defense mechanisms to combat parasite infections, mainly the antimicrobial secretion layer on the gut and cellular and humoral immune mechanisms. Fumagillin, an antibiotic derived from *A. fumigatus,* was recognized as one of the most successful treatments for *N. ceranae* and *N. apis* infection^18,19^. However, accumulation of fumagillin traces in honey and wax is the major challenge^20,21^. Fumagillin blocks the intracellular replication of *Nosema* spp by inhibiting methionine aminopeptidase 2 (MetAP2)^22^*.* Fumagillin and its transformed components that persist in bee products have been linked to human consumption and may have long-term effects on the health and safety of both consumers and apiculturists^23^. So, searching for alternative environmentally safe approaches for controlling the infection by *Nosema* in honeybees is the prospective. Recent studies using bacterial metabolites^24^, organic acids ^25^, essential oils^26^, and plant extracts^27,28^ in addition to dietary supplementation with *Bifidobacterium* and *Lactobacillus* strains have been used^29^, displaying a relative efficiency for combating this disease ^23,30^. As well as, the extracts of *Eleutherococcus senticosus* had a significant activity towards the Nosemosis causing death to honeybee ^31^. As well as natural plant-based products such as propolis have been studied as a substitute for antibiotics in treating *Nosema* infections^32^. Propolis has been shown to reduce *N. ceranae* spore load in infected bees and improve their survival, gland function when given orally, reducing *Nosema* infectivity in honeybees^33,34^. It has antimicrobial, antioxidant, immuno-stimulatory, and anti-inflammatory properties that help in preventing and treatment of various illnesses^35^. Recently, various nutraceuticals including plant extracts such as oregano oil, thymol, carvacrol, naringenin, trans-cinnamaldehyde, tetra-hydro-curcumin, sulforaphane, embelin, allyl sulfide, and hydroxy-tyrosol were used to compact the *N. ceranae* spores in bees, however, these metabolites exhibited a sign of toxicity to bees and human^36^. Interestingly, di-allyl sulfide sourced from garlic demonstrated promising results by decreasing the infection prevalence of *N. bombycis* in silkworm *Bombyx mori* when administered orally ^37^. *Acacia nilotica, Elaeis guineensis*, and *Catharanthus roseus* were selected based on their reported antimicrobial and medicinal properties to be investigated against nosemosis in this study. *Acacia nilotica* exhibits significant antimicrobial activity against various pathogenic bacterial strains like *Porphyromonas gingivalis*, *Aggregatibacter actinomycetemcomitans*, *B. subtilis*, *S. mutans*, *L. acidophilus* and *E. coli*, making it a promising candidate for alternative therapeutic applications^38–42^. *Elaeis guineensis* “oil palm” had a broad-spectrum antibacterial activity to treat wound infections^43–46^. *Catharanthus roseus*, “Madagascar periwinkle”, exhibits significant antimicrobial activity, antioxidant, antigenotoxic, and antimutagenic potential, suggesting its suitability for pharmaceutical applications^47–50^. Research has shown that extracts from different parts of the plants mainly leaves and stems, possess antimicrobial properties against a range of bacterial and fungal strains^51^. Therefore, this study aimed to evaluate the effectiveness of various plant extracts as promising natural products in reducing the spore propagation of (*N. ceranae* and *N. apis*) co-infection and improving the survival rate of inoculated honeybees. Physiological and molecular docking studies were conducted to elucidate the potential possible intermolecular ligand-target interactions of the plant extract on *Nosema* spp.

## Material and methods

### Samples collection, *Nosema* isolation and identification

Forager honey bee samples were collected from outside the hive entrance from bee colonies suspected to be infected with *Nosema* (*N. ceranae* and *N. apis*) “mixed infection” from a beekeeper in December/ 2020 from El Sharkia province in Egypt. Sixty midguts were removed from live adult bees and crushed in sterile distilled water^52^. The homogenate was filtered through a nylon mesh cloth with a pore size of 65 μm. The number of spores in the filtrates was counted by hemocytometer and light microscopy as described by Cantwell^53^, the spores were concentrated by centrifugation at 4,000 rpm for 10 min, then discarding the supernatant. The spore pellets were washed twice in sterile distilled water and centrifugated at 4000 rpm for 10 min. The spores inocula were freshly prepared by diluting the spores with a 50% sucrose solution to get 2 × 10^4^ spores/ml, by the Neubauer counting chamber, as suggested by World Organization for Animal Health (http://www.oie.int/fileadmin/Home/eng/Healthstandards/tahm/2.02.04NOSEMOSISFINAL).

The spores of *Nosema* species were analyzed by phase-contrast microscopy (400X)^54,55^. The *Nosema* spores were stored at 4°C for further use.

### Scanning *electron* microscopy

The *Nosema* spores were centrifuged and collected from forager samples as mentioned above, fixed for 24 h in 5% glutaraldehyde (v/v) in 0.1 M phosphate buffer pH 7.3 prior to SEM analysis. The sample was fixed in Karnowsky fixative for 3 h at 4°C, washed three times in buffer for 10 min at 4 °C, then fixed inosmium tetroxide in 0.1 M cacodylate buffer at 4 °C for 1 h, dried by 50–100% ethanol grade series, coated with gold in an ion sputtering, and then scanned under a scanning electron microscope (SEM) (JEOL JSM-6510/v, Japan)) at Electron Microscope, Mansoura University, Egypt. Data relevant to Scanning Electron Microscopy (SEM) analysis, focused on their morphometric attributes such as length and width of analyzed spores^56^.

### Molecular identification of *Nosema* species

Part of *Nosema* spores that were previously collected, and purified from the abdomen of the infected honeybees undergo DNA extraction^52^. The DNA of *Nosema* species was extracted using i-Genomic BYF DNA Mini Kit (Cat#. 13010348) according to the manufacturer’s instructions. Briefly, the collected *Nosema* spores were re-suspended in MP buffer (100 μl) and 3 μl lyticase solution, vortex for 30 s, incubated for 20 min at 37 °C. The lysate was incubated for 30 min at 65 °C, transferred to spin column in a 2.0 ml collection tube. The extracted DNA was re-suspended in 50 μl of Tris EDTA buffer (pH 8.0) and stored at − 20 °C until use. The identity of *Nosema* species was determined by specific PCR amplification of the 16S rDNA^57^. The PCR reaction was conducted using the extracted genomic DNA as template and primers of 16S rRNA (SSU rRNA) of *N. ceranae* and *N. apis*. The primer set for *N. ceranae* was 5′-CGTTAAAGTGTA GATAAGATGTT-3′ and 5′-GACTTAGTAGCCGTCTCTC-3′, and for *N. apis* 5′-GCATGTCTTT GACGTACTATG-3′and 5′-GACTTAGTAGCCGTCTCTC-3′^52^. The PCR reaction contains 10 μl of 2X PCR master mixture (i-Taq™, Cat. No. 25027), 1 μl of gDNA, each primer (100 pmol), the reaction was completed to 25 μl with sterile distilled water. The PCR was programmed to initial denaturation at 94 °C for 2 min, followed by denaturation at 94 °C for 30 s, annealing at 51 °C (*N. ceranae* primers) and 60 °C (*N. apis* primers) for 55 s, extension at 72 °C for 30 s for 35 cycles, and final extension at 72 °C for 2 min^58^. The amplicons were analyzed by 2% agarose gel in 1X TBE buffer, compared to the negative control PCR reaction at 5 V/cm. The size of the obtained products was evaluated by comparison with the Gene Ruler 100 bp Plus DNA Ladder (Fermentas) molecular size marker, confirming species. Ethidium Bromide was added to the gel at 0.5 μg/ml to visualize the resulting DNA fragments of *Nosema* spp. Electrophoresis results were achieved using the GelDoc (Bio-Rad) gel documentation system.

The amplicons were visualized, purified, and sequenced by Applied Biosystems Sequencer, HiSQV Bases, Version 6.0 with the same primer. The obtained 16 S rRNA gene sequences were non-redundantly BLAST searched with sequences of NCBI database. The FASTA sequences were imported into MEGA X software, aligned with Clustal W muscle algorithm, and the phylogenetic tree was constructed with neighbor-joining method with 1000 bootstrap replication.

### Quantitative real-time PCR (qRT-PCR) for *Nosema* quantification

The relative concentration of *Nosema (Vairimorpha)* spores in the most effective treatments in bioassay was determined by Real-Time PCR assay depending on SYBER chemistry and species–specific primers. The genomic DNA of *Nosema* from five bees for each treatment was extracted as described above, used as PCR template. The PCR reaction mixture contains 10 μl Xpert Fast SYBR master mix with low Rox (1X), 1 μl of each primer, 2 µl of genomic DNA and 6 µl sterile distilled water. The qRT-PCR (Bio-Rad’s CFX96 Real-Time PCR) was programed to initial denaturing at 94°C for 2 min, 40 cycles at 94°C for 20 s, annealing at 60°C for 30 s, with final extension at 72°C for 30 s. The primers set were 5′-GACGGAAGAATACCACAAGGAG -3′, 5′- CGGCCATGCACCACTATTA -3′ for *N. ceranae* (designed by PrimerQuest™ Tool depending on *Nosema* ceranae 16S ribosomal RNA, NCBI Reference Sequence: XR_002966746.1 and checked by PRIMER BLAST), and 5′-GCATGTCTTTGACGTACTATG-3′, 5′-GACT-TAGTAGCCGTCTCTC-3′ for *N. apis*^52^ & primers for β-actin 5′- ATGCCAACACTGTCCTTTCTGG-3′, 5′- GACCCACCAATCCATACGGA-3′ (designed by this study). Negative controls for the PCR reactions were used. Triplicates for the reactions were used. The fungal loads were analyzed by 2^-ΔΔ*CT*^ method, as a relative quantification strategy for quantitative real-time polymerase chain reaction (qPCR) data analysis^59^. The difference between the threshold cycle (Ct) number for honey bee β-actin and that of *Nosema* sp. of interest was calculated^60^.

### Preparation of the plant extracts

Some medicinal plants were selected based on their ethnological and biological properties, namely *Acacia nilotica*, *Elaeis guineensis* (Palm fruits) and *Catharanthus roseus*^38,39,41,44,45,47,49^. *Acacia nilotica* and *Catharanthus roseus* were collected from the Campus of Zagazig University, and identified by Dr. Marwa El-Demerdash, Faculty of Science, Zagazig University, Egypt. The samples of *Elaeis guineensis* (Palm fruits) were collected from Aswan Province, Egypt, and identified by Prof. Monier Abd El-Ghani, Cairo University, Egypt. A voucher specimen of *A. nilotica, C. roseus,* and *E. guineensis* has been deposited at Botany Department Herbarium, Faculty of Science, Zagazig University (BDH-ZU), with deposition numbers BDH-ZU-AN10, BDH-ZU-CR11 BDH-ZU-EG12, respectively. The plant materials were collected according to the relevant institutional, national and international guidelines and legislations.

The air dried plant parts (Leaves, stem, and flower of *Acacia nilotica* and *Catharanthus roseus* or peeled Palm fruits which divided into two parts for extraction, nuts(exocarp) and retaining flesh (mesocarp, endocarp, embryo) (100 g) were macerated in 500 ml methanol, and incubated for 3 days at 30°C under shaking condition in dark. The mixtures were filtered to remove the particles, and the samples were concentrated by the rotary evaporator at 40°C, till oily residues. The dried crude extracts were weighed, dissolved in 500 µL methanol, and stored as stock with known concentration in glass vials at 4 °C until used for further studies.

### The preliminary toxicity test of the plant extracts to the honeybees (*Apis* mellifera L.)

The toxicity of the plant extracts on adult worker healthy honeybees (*Apis mellifera* L.) “uninfected” was assessed under laboratory conditions after an exposure period of 2 days of uninfected bees to plant extracts at the Laboratory of Pest Physiology, Plant Protection Research Institute, Zagazig, Sharkia Governorate. The European honeybee (*Apis mellifera*), is neither an endangered or protected species, the study was conducted in June-July 2020. Frames with sealed broods from healthy colonies, which were identified as *Nosema*-negative through a monthly disease survey^53^, were removed from bee colonies without nurse bees. The frames were then placed in individual mesh-walled cages and stored in an insect growth chamber at 34 ± 1°C and 55 ± 5% relative humidity overnight.

The frames were incubated for 24 h, to allow newly emerged bees to roam on the whole frame consisting of brood, pollen, and honey to acquire gut microbiota, emerged worker bees were collected from the frames to be used for subsequent *Nosema* spores inoculation. The negative status of *Nosema* infection in newly emerged bees was confirmed using a hemocytometer and light microscopy^53^, to ensure the bees were free of *Nosema*.

#### Treatments

Three cups housing 11 bees each were used for each group, for a total of 36 cages (four treatment groups/three concentrations). Bees fed ad libitum with 50% sucrose solution containing 0.05%, 0.1%, and 1% (w/v) of each (previously prepared) plant extract and water in 15 ml drip feeders for two days. On the third day of treatment, all groups fed on ad libitum only with 50% sucrose solution without any plant extract. Mortality and behavioral abnormalities were monitored, and dead bees were counted and removed daily till the 5th-day post-treatment (period of the experiment) or acute for 3 days only. The toxicity and palatability of the plant extract blend on *A. mellifera* were evaluated before the beginning of the experiment. The adequate concentration of the plant extract blend was selected. For that reason, honeybees were treated with 0.1% (w/v).

### Bioactivity of the plant extracts against *Nosema* species infecting honeybee

The acute and chronic oral toxicity of the four tested plant extracts on the adult worker honeybees under laboratory conditions were assessed. Briefly, the honeybee workers were collected from the hive entrance of an apiary and checked for *Nosema* infection. Thirty bees were tested to ensure that none of them had any detectable *Nosema* spores. The bees were then placed in plastic cup cages and kept in dark incubator at 33 ± 2 °C, with relative humidity 60 ± 5%. To simulate the hive environment, a small piece of wood and a small wax foundation sheet were placed on top of each cage. Pores were created in the bottom of the cups to maintain hygiene, each cup was equipped with 10 ml syringe filled with a 50% sucrose solution for the bees feeding. The syringes were modified to allow easy access to the food while preventing any leakage. Previously extracted spores were quantified using a hemocytometer and adjusted to 20,000 spores/ml, in 50% sugar syrup. The worker bees were starved for 2 h before being infected by the direct group feeding with 50% sugar syrup mixed with the extracted spores (20,000 spores/ml) in drip feeders for 2–4 h. The artificially infected adults were transferred randomly to new three plastic top-feeder rearing cups. Three replicates were run in each treatment group for a total of 33 bees tested per treatment and a total of 24 cages (four treatment groups/two concentrations) for the experiment. Bees fed ad libitum with 50% sucrose solution containing 0.1% and 1% (w/v) of each plant extract and water in 10 ml drip feeders for two days in addition to a small thin layer of candy with pollen mixed with 50% sugar syrup containing plant extract with the same conc. Mortality and behavioral abnormalities were monitored, and dead bees were counted and removed daily throughout 11 dpi excluding bees that died within 2 dpi, as this is typically due to handling and inoculation stress and not *Nosema* infection.

### Determination and quantification of *Nosema* (*Vairimorpha*) spores load after plant extracts treatments

The spores of *Nosema* ceranae were microscopically quantified by Neubauer counting chamber, and by the RT-qPCR, as described above. The same treatments (as the previous bioassay) were repeated, and infected bees were exposed to 2 days of plant extracts (0.1%) treatment under laboratory Conditions. Five bees from each treatment group were randomly collected on days 5 and 9 after infection to be examined for *Nosema* spores. The spore was extracted with the same method described before. After centrifugation, the supernatant was poured, and the pellet was used for counting. One ml of distilled water was added to the pellet and was vortexed (so 200 μl per bee). These pellet solutions from each sample were divided into two groups; 200 μl, was used to count *Nosema* spores under a light microscope with 400 × magnification with Neubauer slide. Infection levels per bee were determined ^52,53^ and other, 800 μl for determining the level of *Nosema* infection by qPCR.

### Biochemical studies

The physiological effects in adult honeybees induced by *Nosema* infection and plant extract treatments were evaluated at a concentration of 0.1% (w/v) and a 48-h feeding period. The evaluation was conducted at various time points: 5-dpi, 9-dpi, and 3-dpt. This was accomplished through the examination of specific physiological characteristics related to neural activity (acetylcholinesterase (AChE)), immunity (alkaline phosphatase (ALP), involved also in metabolism), and defense against oxidative stress and detoxification (glutathione-S-transferase (GST)). A double-beam ultraviolet/visible spectrophotometer (spectronic-1201, Milton Roy Co., USA) was used to measure the absorbance of colored substances.

The insect homogenates were prepared by homogenization in distilled water, followed by centrifugation at 8000 rpm for 15 min^61^. The bee homogenates were stored at 4 °C, till use. The activity of glutamic pyruvic transaminase (GPT) and glutamic oxaloacetic transaminase (GOT) were determined calorimetrically as described by Reitman & Frankel^62^. The enzyme activity was expressed by U /g body weight.

The digestive enzymes were determined according to Ishaaya and Swirski^63^ with a slight modification using sucrose and trehalose as substrates for invertase (EC 3.2.1.26) and trehalase (EC 3.2.1.28), respectively). The enzyme activity was expressed as μg glucose released /min/g fresh weight. The activity of alkaline phosphatases was determined using p-nitophenyl phosphate as substrate as described by Powell and Smith^64^. The Proteolytic activity (Protease, EC 3.4.21.112) was measured as described by Tatchell et al.^65^, with some modifications, by measuring the increase in free amino acids split from substrate protein (albumin), during one-hour incubation at 30 ˚C. Amino acids were colorimetrically assayed by ninhydrin reagent at 570 nm^66,67^.

The activity of glutathione S-transferase (GST) was determined based on the conjugation of reduced glutathione (GSH) with 1-chloro 2,4-dinitrobenzene (CDNB), and the conjugate, S-(2,4-dinitro-phenyl)-L-glutathione was measured^68^. The activity of acetyl-cholinesterase (AchE) was measured using acetylcholine bromide (AchBr) as substrate at 515 nm^69^. The total proteins were determined by the Coomassie Brilliant blue G-250 dye^70^.

### Chromatographic analysis for the bioactive compounds of the most effective extract

The most potent extract was selected and subjected to GC–MS analysis to identify the compounds present in the crude extracts^71^. GC–MS/MS analysis of the fungal crude extracts was performed using an Agilent Technologies 7890A gas chromatograph interfaced with a mass-selective detector (MSD, Agilent 7000). The chromatograph was equipped with a polar Agilent HP-5 ms (5% phenyl methyl poly siloxane) capillary column (30 m × 0.25 mm i.d. and 0.25 μm film thickness). A volume of 1 μL of the sample was injected. Helium was used as the carrier gas with a linear velocity of 1 mL/min. The injector and detector temperatures were set at 200 ºC and 250 ºC, respectively. The MS operating parameters were as follows: ionization potential of 70 eV, interface temperature of 250 ºC, and acquisition mass range of 50–800. To identify the bioactive compounds present in the extracts, their mass spectra and retention time were compared with those of authentic compounds on the NIST (National Institute of Standards and Technology) and WILEY libraries. Additionally, the fragmentation patterns of the mass spectral data were compared with those reported in the literature. The name, molecular weight, and structure of the components of the test material were determined.

### Molecular docking analysis

Molecular docking aimed to illustrate the virtual mechanism of binding; selected compounds in the most effective extract as an anti-nosemosis agent with predicted active sites in targeted species of interest (Nosema) modeled domain. All the component structures were obtained from the PubChem database. To further assess their interactions with the targeted modeled domain (by using the I-TASSER server) on the molecular level through conducting molecular docking simulations. After that, the format was converted from pdb to pdbqt by Open Babel (Version 2.3.1) software^72^. The target receptor was prepared by removing water and other co-crystalized ligands and adding polar hydrogen atoms. Also, the grid box was adjusted to target the entire molecule with a grid spacing of 1Å between the set points. The grid box was centered at 57.876, 60.851, and 57.846 at points 62, 54, and 54 on x, y, and z dimensions. Docking simulations and binding affinity estimation were performed with AutoDock Vina^73^. To better understand the ligand–protein interaction, BIOVIA discovery studio was employed to visualize the molecular interactions and detect the chemical bonds formed during the binding^74^.

### Statistical analysis

All the experiments were conducted with three biological replicates. Statistical analyses were performed using Costat software^75^ program. The obtained results of mortality, *Nosema* spore count, QPCR, and biochemical data were subjected to One Way ANOVA, and Tukey HSD to determine if there were significant differences between means (groups) when compared at P < 0.05.

## Results

### *Nosema* spore detection and identification

**Fig. 1:**
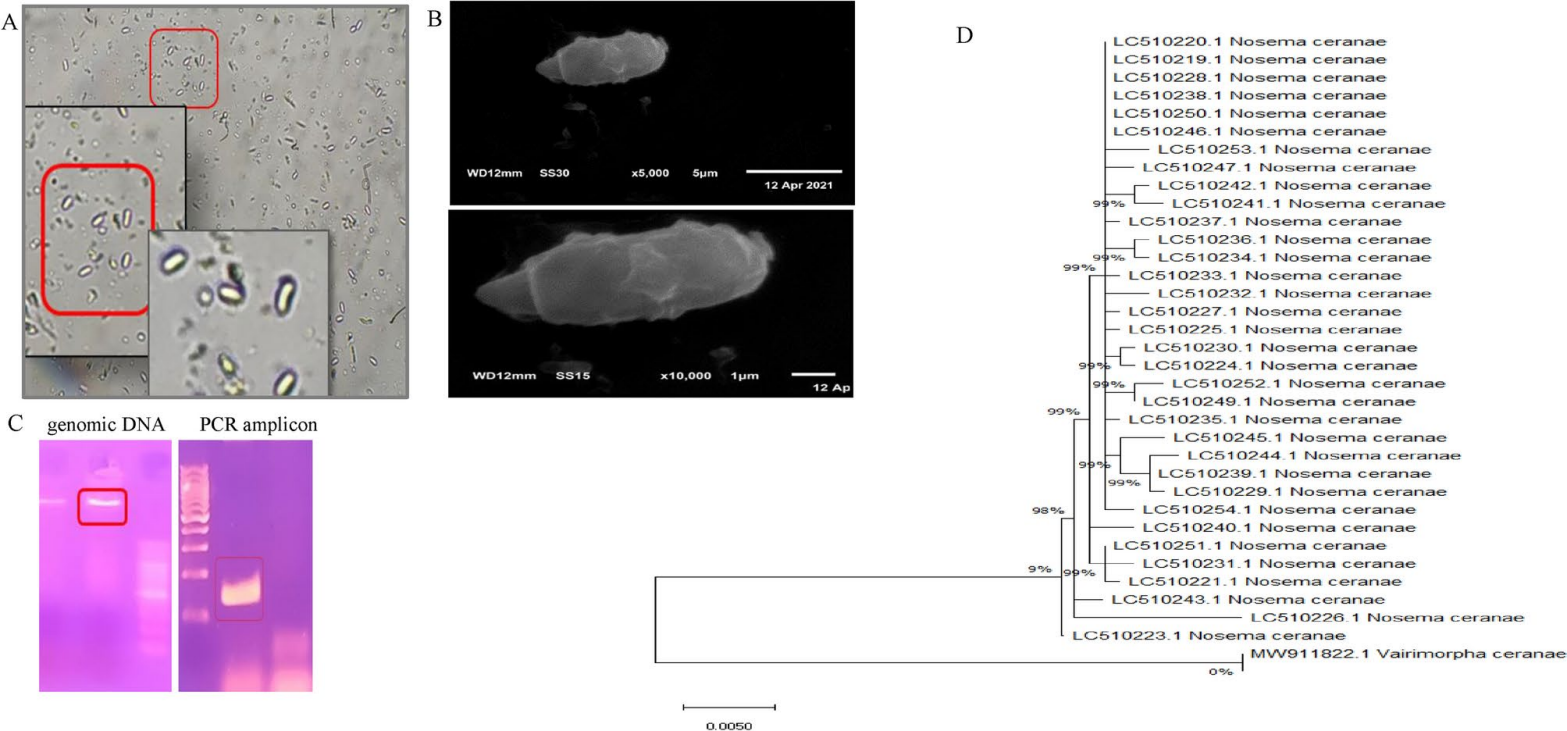
Morphological identification and molecular confirmation of *Nosema* species. (**A**) *Nosema* spores in the macerated abdomens suspension of adult honeybees by phase-contrast microscopy at 400 X. (**B**) Scanning electron microscope of *Nosema* (*Vermiphora*) *ceranae* at 5000 × and 10,000 x. (**C**) Genomic DNA and PCR amplicons of *Nosema ceranae.* The PCR products of 16 S RNA of 145 bp, with the specific primers for *N. ceranae*. (**D**) The phylogenetic tree of 16S RNA sequence of *Nosema ceranae*, with the database deposited sequences of *Nosema* species by Maximum Likelihood method (Tamura, 2007).

The adult honeybee samples from the Egyptian apiary in El Sharkia province were collected with biological signs of infection with *Nosema* species. The spores of *Nosema* were collected from the abdomen of the bees and visualized by light microscopy and scanning electron microscope (Fig. 1). The spores of *Nosema* species were oval to rod-shaped as shown from the microscopic analysis. For more confirmation and to determine the species, the PCR amplicon product was visualized on agarose gel. DNA was extracted from spores and amplified with the mentioned primer pair derived from 16SrRNA of *Nosema* spores. The amplified DNA fragment revealed that the isolation of DNA from spores was successfully performed (Fig. 1). The 16SrRNA gene has been successfully applied to differentiate between *Nosema* species according to expected size of constructed amplicons. After visualizing the PCR product on a 2% agarose gel electrophoresis, *N. apis* was detected at 224 bp, and *N. ceranae* at 145 bp, allowing for differentiation between the two species.

### Toxicity of the plant extracts to the natural honeybees

The toxicity of the different methanolic extracts of *Acacia nilotica*, *Elaeis guineensis* 1(fruit endocarp), *E. guineensis* 2 (fruit exocarp), and *Catharanthus roseus* towards the adult honeybees (worker bees) were assessed that were not infected or treated under laboratory conditions (at 34 ± 1°C and 55 ± 5% RH). The plant extracts at different concentrations (0.05%, 0.1%, and 1%) were amended to the nutrients of sucrose solution of the adult healthy honeybees (worker bees) as control (uninfected bees). The cumulative mortality and vitality of the bees were recorded daily over three successive days. The mortality data of the bees in response to the different concentrations of the plants were reported in Table 1. Obviously, the toxicity of the tested plant extracts was proportionally increased with the concentration and days per treatment of the honeybees. On the first day of treatment, the extracts of *A. nilotica*, *E. guineensis* 1 (fruit endocarp), *E. guineensis* 2 (fruit exocarp) had no effect on the honeybees, unlike to the slight effect by the *Catharanthus roseus*. By the 2nd and 3rd dpt, the extract of *A. nilotica* at 0.05 and 0.1% had no toxic activity towards the honeybees, as revealed from the cumulative mortality, however, at 1% the extract exhibits a relative higher mortality rate of bees by about 3.8% and 7.6%, respectively. The extracts of *E. guineensis* 2 still had zero cumulative mortality, while *E. guineensis* extract 1 and *C. roseus* had a low and non-significant cumulative mortality of 3.03%. On the 3rd day post-treatment, feeding of the bees on plant extracts of 0.05%, and 0.1% in 50% sucrose solution has no impact on the bees survival, however, at 1.0%, the extracts had a signs of cumulative mortality to the bees. At 1.0%, there was a significant decrease in the cumulative mortality 3.03, 3.03, and 9.09% for *E. guineensis* extract 1, *E. guineensis* extract 2, and *C. roseus*, respectively, compared to the uninfected control. However, at concentration of 1% (10 mg/ml), the four extracts causing a significant decrease on the count of live bees, with a substantial increase on the mortality rates by about 7.69%, 38.4%, 50%, and 76.92% for *A. nilotica, E. guineensis* extract 1*, E. guineensis* extract 2 and *C. roseus*, respectively, at the 3^rd^ dpi. These results confirm the safety of the tested extracts at concentrations of 0.05%. Particularly, *A. nilotica* extract was found to be the safest one at concentrations of 0.05% and 0.1% up to 3dpt, however, *C. roseus* was found to be the most toxic one at concentrations 0.1% and 1%. From the obvious behavioral, bees treated with *E. guineensis* of 0.1% exhibited neural abnormalities and nervous behavior, while bees treated with *C. roseus* showed shrinkage and dryness, which are typical signs of chemical toxicity in insects. The flight of the treated bees was not affected in any of the treatments, except for those treated with *C. roseus*, which were significantly affected. Based on these findings, a concentration of 0.1% was selected for further antimicrobial activity screening assays.

**Table 1:** Accumulative mortality from plant tested extracts treatment of untreated uninfected honeybees under laboratory conditions (at 34 ± 1 °C and 55 ± 5% RH):

Treatment	Accumulative mortality %									
	Time	1dpt			2dpt			3dpt		
	conc	0.05%	0.1%	1%	0.05%	0.1%	1%	0.05%	0.1%	1%
*Acacia nilotica* extract	0^a^	0^a^	0	0^a^	0^a^	3.8	0^b^	0^b^	7.69	
*Elaeis guineensis* 1(fruit flesh endocarp) extract	0^a^	0^a^	0	3.03^a^	3.03^a^	11.5	3.03^ab^	3.03^b^	38.4	
*Elaeis guineensis* 2 (fruit exocarp) extract	0^a^	0^a^	3.8	0^a^	0^a^	11.5	0^b^	3.03^b^	50	
*Catharanthus roseus* extract	0^a^	3.03^a^	7.6	3.03^a^	3.03^a^	42	6.06^a^	9.09^a^	76.92	
Untreated uninfected control	0^a^	0^a^	0	0^a^	0^a^	3.8	0^b^	0^b^	7.69	
Lsd		….		…	….		….	6.03		
P	n.s	n.s		n.s	n.s		n.s	(0.041)*		

Means values are given for mortality (%).

The mean values followed by different letters a, b, c within the same column is significantly different (ONE Way ANOVA, LSD test, *p* ≤ 0.05). LSD: the least significant difference. n.s: non-significant. (*) significance. *** means highly significant.

Plant materials toxicity and Palatability of treatments (Toxicological studies and preliminary bioassay results):

Acute toxicity effect of Plant tested extracts on untreated uninfected honeybees under laboratory conditions (at 34 ± 1 °C and 55 ± 5% RH):

### Effect of the plant extracts on honeybees artificially infected with *Nosema* spp

The acute and chronic oral toxicity assays were conducted to evaluate the effects of the four tested plant extracts at 0.1% and 1% on the adult worker honeybees infected with *Nosema* spores under laboratory conditions at five different time points (3 days, 5 days, 7 days, 9 days, and 11 days). After each time, the corresponding mortality percentages were calculated. The results showed that there was a significant difference in mortality between the treatment groups and the control after three days of infection, however, no significant difference was observed at 5 days post-infection (Table 2). Furthermore, there were highly significant differences in mortality between all treatment groups and their respective controls at 7 days, 9 days, and 11 days post-infection. This could be attributed to the effects of the plant extract on both bee survival and the propagation of *Nosema* spores, which might have a negative effect of the spore growth of infected bees. At 3 days post infection, the acute toxicity of all treatments was zero, except for *C. roseus*, which had the highest acute toxicity among the four treatments with a mortality rate of 6.06%. After 11 days of infection, the toxicity of the plant extracts was assessed, *A. nilotica* extract had the lowest mortality rate of 18.18%, revealing their quite safety and efficiency against *Nosema* infection compared to the control group with a mortality rate of 30.3% (Table 2). At 0.1%, *C. roseus* extract causes mortality by about 33.3% to the bees. Both extracts of *Elaeis guineensis* had the highest mortality rates to the bee individuals, with about 48.48% and 42.42%, respectively, revealing the affordable toxicity of these extracts to the bee individuals. So, *E. guineensis* extracts could have highly cytotoxic compounds to the bees, in addition to their antimicrobial activity, so, these plant extracts were excluded from the further anti-parasitic assay. Feeding of the bees at 1% (10mg/ml) of the tested extracts increase the percentage of bees’ mortality to 30.30%, 42.42%, and 72.73% and 100%, respectively (Table 2). So, the lowest toxicity of the extracts to the bees can be arranged in an ascending manner; *A. nilotica* extract, *E. guineensis* 2 (fruit exocarp), *E. guineensis* 1 extract (fruit fleshly endocarp) and *C. roseus* after 7-day post-infection, compared to untreated infected control which recorded mortality percent 12.12%.

**Table 2:** Mean bees mortality ± SE (%) at concentration level 0.1% (1 mg/ml) of different plant extract treatments.

Treatment	N	Mean ± SE mortality rate at concentration level 0.1%				
		3dpi	5dpi	7dpi	9dpi	11dpi
*Acacia nilotica* extract	33	0^b^	0^a^	12.12^ab^	18.18cd	18.18cd
*Elaeis guineensis* extract1	33	0^b^	3.03^a^	18.18^a^	39.39^a^	48.48^a^
*Elaeis guineensis* extract2	33	0^b^	3.03^a^	15.15^a^	30.30^ab^	42.42^ab^
*Catharanthus roseus* extract	33	6.06^a^	6.06^a^	18.18^a^	27.27^bc^	33.33^b^
Untreated Infected control	33	0^b^	0^a^	6.06^bc^	15.15^d^	30.30^bc^
Untreated uninfected control	33	0^b^	0^a^	0^c^	0 ^e^	6.06^d^
Lsd		3.81	……	6.6	9.33	12.64
P		0.0228*	.3149 ns	0.0003***	.0001***	.0001***

Bioassay and antimicrobial activity against Nosema infection under lab conditions:

3.a. Impact of the tested Plant Extract on HoneyBees Artificially Infected with *N. sp* under lab Conditions: combined effect of tested extracts in addition to nosema infection on survival of bee.

### Microscopic quantification of *Nosema* spores of honeybees in response to the plant extract treatments

*Nosema* species spores were microscopically quantified by the Neubauer slide to assess the in vivo anti-microsporidian activity of the tested plant extracts. Infection level development per bee (exposed to 2 × 10^4^ spores/ml), was monitored as the number of spores present in the abdomen of surviving honeybees at the time points 5 dpi and 9 dpi. The number of *Nosema* spores in the honeybees treated with the different plant extracts at 5 and 9 days of the artificial inoculation, were quantified (Table 3). Additionally, the difference in spore counts between the treatments and the control was higher at 9 dpi compared to 5 dpi. The extract of *E. guineensis* endocarp 2 extract at 9 dpi, had a similar spore count to the infected control that may be due to the spread of *Nosema* infection in the untreated infected control over time. The extracts of *A. nilotica* exhibited the lowest spore load per bee at 5 dpi, with 23 × 10^5^ spores per bee, compared to the control’s 37 × 10^5^ spores, indicating it as the most effective extract in reducing *Nosema* spores by 37.8%. While, the other extract showed a slight decrease in the spore count per bee of *C. roseus*, *E. guineensis* 2 extract (fruit exocarp), and *E. guineensis* 1 extract (fruit endocarp), with recorded values of 27 × 10^5^, 29 × 10^5^ and 34 × 10^5^, respectively. At 9 days post-infection, *A. nilotica* and *C. roseus* extracts showed the highest reducing effect on *Nosema* spores in the infected bees as shown from the spores count 38 × 10^5^ spores, 35 × 10^5^ spores per bee, respectively, compared to the untreated control (52 × 10^5^ spores per bee). The results suggest that *A. nilotica* had the most powerful activity against *Nosema* species, followed by *C. roseus*. At 9dpi, the spore count was increased but remained lower than that of the untreated infected control. Furthermore, the results indicated that the two-day treatment of the plant extracts displayed a short-term effect, highlighting the importance of repeating the treatment dose after a few days. The infectivity level was highest on 9th day post infection, while the lowest infection was observed on the 5th day of post infection, compared to the infected untreated controls, *p* < 0.0001 (Table 4). So, from the microscopic examination, significant fungicidal activity was reported for the extracts of *A. nilotica* and *C. roseus*, as revealed by the strong reduction in the spore counts of *Nosema* species.

**Table 3:** Spore count under microscope.

Day/Methanolic extract		*Acacia nilotica*	*Catharanthus roseus*	*Elaeis guineensis* 3	*Elaeis guineensis* endocarp2	Untreated infected control	*P*	LSD
5-dpi	spore count (× 10^5^)	23 ^b^ ± 4.5	27 ^ab^ ± 5	29 ^ab^ ± 1	34 ^ab^ ± 6	37 ^a^ ± 4	0.0211*	806,450.8
	(reduction/increase) %	− 37.84	− 27.03	− 21.62	− 8.11	0		
9-dpi	spore count (× 10^5^)	35.3 ^c^ ± 1.5	38.8^c^ ± 1.5	43.5 ^b^ ± 1.5	53.5 ^a^ ± 2.5	52.3^a^ ± 2	< 0.0001***	298,936.2
	(reduction/increase) %	− 32.50	− 25.81	− 16.82	2.29	0		

Values given are mean ± SD.

The mean values followed by different letters a, b, and c within the same row are significantly different (ONE Way ANOVA, LSD test, p ≤ 0.05).

*Significant difference. ** means highly significant.

LSD: the least significant difference. ${\text{(reduction}}/{\text{increase) }}\% = \frac{{{\text{treatment}} - {\text{control}} }}{{{\mathrm{control}}}}*100$

**Table 4:** Quantification of *Nosema apis* and *N. ceranae* by RT-qPCR.

Day/treatment		*N. apis*		*N. ceranae*	
		^ΔΔC^T*	Fold change**	^ΔΔC^T	Fold change
5-d.p.i	*Acacia nilotica*	0.9 ± 0.3	0.54	1.7 ± 0.2	0.31
	*Catharanthus roseus*	1 ± 0.4	0.50	1.55 ± 0	0.34
	infected untreated control	0 ± 0	1.00	0 ± 0	1.00
	uninfected untreated control	1.4 ± 0	0.38	2.55 ± 0.3	0.17
	P	.0008***		.0000***	
	LSD _0.05_	0.47		0.33	
9-d.p.i	*Acacia nilotica*	2.17 ± 0.4	0.22	3.55 ± 0.55	0.09
	*Catharanthus roseus*	1.77 ± 0.4	0.29	2.1 ± 0.5	0.23
	infected untreated control	0 ± 0	1.00	0 ± 0	1.00
	uninfected untreated control	3.77 ± 0.6	0.07	5.75 ± 0.75	0.02
	P	.0000***		.0000***	
	LSD _0.05_	0.77		0.99	

*ΔΔCT (ΔCT nosema sp. treated—ΔCT Untreated).

**Fold change = 2^(-ΔΔCT).

### qPCR quantification of *Nosema* spores infecting honeybees in response to plant extracts

**Fig. 2:**
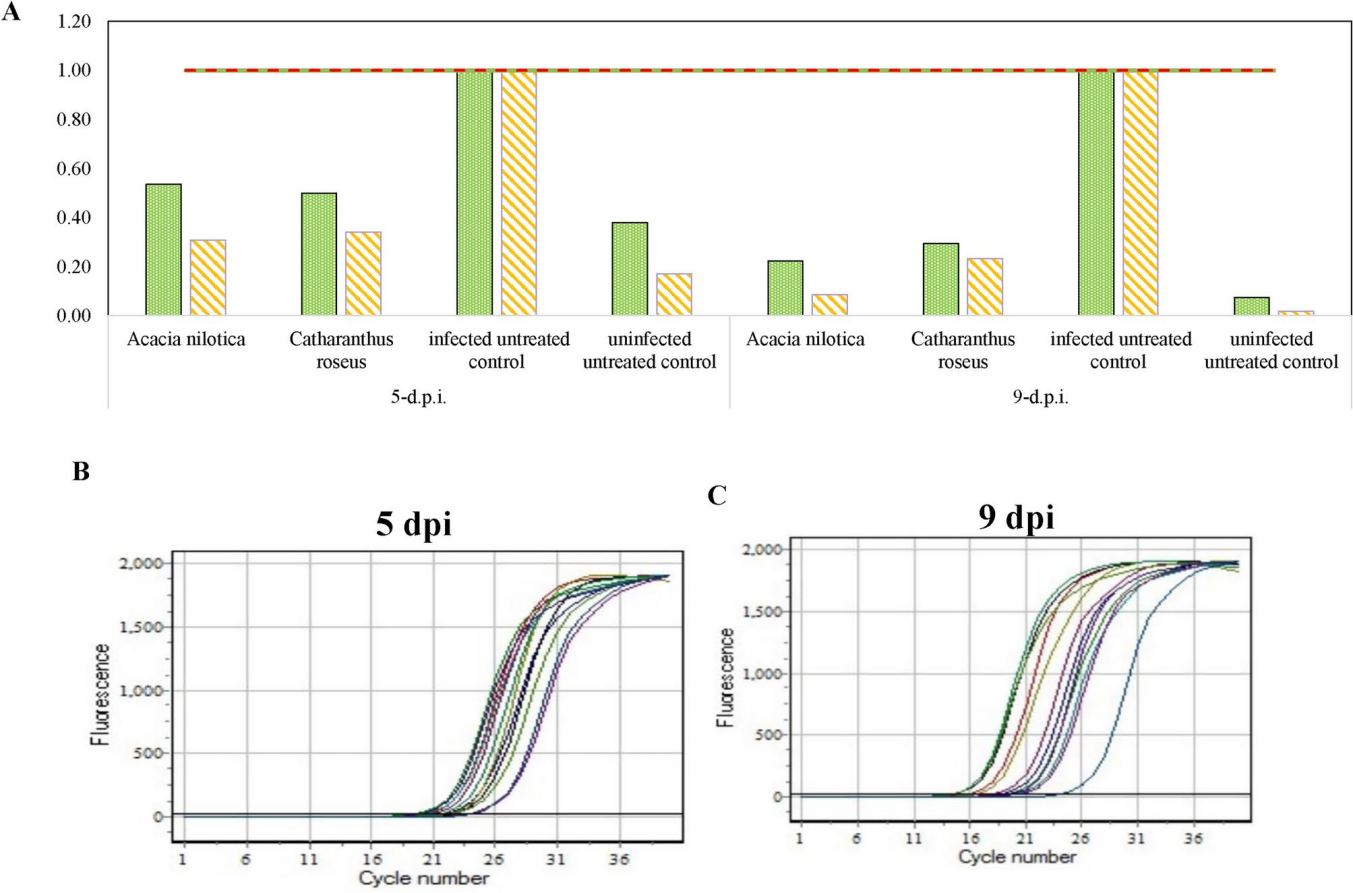
Quantification of *Nosema* spores load by qPCR in response to the extracts of *A. nilotica* and *C. roseus* at 0.1%. A, Fold of change of *Nosema apis* and *Nosema ceranae* in the abdomen of honeybees in response the plant extracts (**A**). The amplification curve of qPCR for *N. ceranae* at 5 days post infection (**B**) and 9 days post infection (**C**) in response to the plant extract.

The load of *Nosema* species was microscopically quantified in honeybees in response to different plants extracts, then further confirmed by the qPCR analysis compared to the untreated controls. The most effective plant extracts (*A. nilotica* and *C. roseus*) in reducing *Nosema* spores were selected for more confirmatory analysis by qPCR. The presence of *N. apis* and *N. ceranae* was determined in response to treatment with *A. nilotica* and *C. roseus*, negative (uninfected untreated), and positive (untreated infected) control samples, based on the amplification threshold and melting curves obtained from qPCR results (Fig. 2). Anti-microsporidian effect of the plant extracts on *Nosema* spores load of honeybee was evaluated from the Δ^CT^ values of treated samples compared to the untreated samples (positive control for *Nosema* species), after 5-day and 9-day post-treatment (Table 4). From the melting temperature curve (Fig. 2), the Tm values were 77.9°C and 84.2°C for *N. ceranae* and *N. apis*, respectively, while was 79.8 Tm for housekeeping gene. The fold change in parasite DNA abundance of the treatments and control samples was calculated from the 2^(-ΔΔCt)^ value. The qPCR data analyzing the gene abundance levels of *N. apis* and *N. ceranae* in the treated and untreated honeybees after 5 and 9-days post-infection (dpi) were illustrated in Table 4. From the qPCR analysis, a notable difference in the spores’ load of *N. apis* and *N. ceranae* among the treated samples compared to the untreated positive control. A relative abundance in parasite load less than 1 in all treatments compared to control indicating the effectiveness of treatments in reducing *Nosema* spore load and infection level. At 5-day post infection, *A. nilotica* and *C. roseus* extracts showed show decreased ΔΔCT values compared to infected untreated controls, indicating a reduction in gene levels of *Nosema* species. The extracts of *A. nilotica* had a significant activity against *N. ceranae* as revealed from the *p*-values (*p* < 0.0001***), as well as from the LSD values provide insights into the variability within treatment groups. At 9 d.p.i., the tested plant extracts displayed a significant reduction in the abundance of *Nosema* species as revealed from the ΔΔCT values compared to infected untreated controls, suggesting the strong suppression of *Nosema* spp abundance. Statistical analysis reveals a significant difference between treated and untreated groups for both *N. apis* and *N. ceranae* (*p* < 0.0001***). Overall, upon using the extracts of *A. nilotica* and *C. roseus* in infected honeybees, the growth and abundance of *Nosema* species were strongly suppressed. So, from the microscopic quantification and qPCR analysis, the abundance of *Nosema* species infecting the honeybees was strongly reduced upon using the methanolic extracts of *A. nilotica* and *C. roseus,* particularly in *A. nilotica* treated samples, which appears to be with no obvious toxicity to the honeybee as revealed from the mortality rate at tested concentration. Increasing treatment exposure duration is expected to improve outcomes in future assessments.

### Biochemical changes of the natural uninfected honeybees in response to the tested plant extracts

The effect of the tested plant extracts on the biological properties of uninfected honeybee was assessed, by implementing the extracts at 0.1% concentration for 48 h feeding period, then various biochemical parameters were measured mainly total proteins, activity of GPT, GOT, AchE, ALP and GST as described above. The biochemical parameters of the uninfected honeybee in response to the plant extracts were summarized in Table 5. Overall, an obvious disturbance of the total protein in the uninfected control honeybees in response to the plant extracts was observed, compared to the untreated uninfected control. Among the tested plant extracts, *A. nilotica* extract had the mild effect on the total protein contents of honeybees. A strong decrease in the total proteins was observed upon using *E. guineensis* extracts (1) and (2) at 0.1% (3 days post-treatment) by 36.3 and 27.8%, respectively. The protein content of uninfected adult bees has been highly increased upon using of *C. roseus* extracts (43.06%), compared to control.

**Table 5:** Effects of four tested plant extracts on biochemical parameters in uninfected bees on the third-day post-treatment.

Plant Methanolic Extract treatments	Total proteins (mg protein/g body weight)		GPT (mU/g. b. wt.)		GOT (U/g. b. wt.)		AChE (µg AchBr /min/g. body weight)		ALP (U/g. b. wt.)		GST (m mole substrate conjugated /min/g. b. wt.)	
	Conc	RC %	SA	RA%	SA	RA%	SA	RA%	SA	RA%	SA	RA%
*Acacia nilotica*	24.2 ± 0.8 ^b^	5.99	3970.95 ± 36.5 ^a^	16.3	67.03 ± 1.83 ^c^	− 18.90	111.33 ± 11.02 ^d^	− 9.73	784 ± 38.5 ^b^	− 39.38	45 ± 2.65 ^a^	− 1.47
*Elaeis guineensis*1	14.53 ± 0.2^c^	− 36.3	3639.41 ± 105.7^b^	6.67	59.82 ± 5^c^	− 27.63	445.33 ± 47.38 ^a^	261.09	376.67 ± 14.29^c^	− 70.88	24.33 ± 3.21^b^	− 46.73
*Elaeis guineensis* 2	16.47 ± 0.6^c^	− 27.8	2510.38 ± 98.5^d^	− 26.4	132.7 ± 7.8^a^	60.59	363 ± 32.51^b^	194.33	347 ± 15.7 ^c^	− 73.17	29.67 ± 2.52^b^	− 35.03
*Catharanthus roseus*	32.67 ± 1.6^a^	43.06	3478.12 ± 79.0 ^c^	1.9	39.78 ± 0 ^d^	− 51.87	206.33 ± 20.26 ^c^	67.30	603 ± 14.7 ^b^	− 53.38	42 ± 2 ^a^	− 8.04
Untreated uninfected control	22.83 ± 1.2^b^	0	3411.81 ± 43.78^bc^	0	82.65 ± 3.67 ^b^	0	123.33 ± 20.82 ^d^	0	1293.33 ± 170.3^a^	0	45.67 ± 3.05 ^a^	0
LSD	1.88		141.92		8.26		53.14		143.68		4.94	
P _0.05_	> .0001***		> .0001***		> .0001***		> .0001***		> .0001***		> .0001***	

Values given are the mean ± SD.

Means, within column, bearing different letters are significantly different (ONE Way ANOVA, Tukey’s HSD test, p ≤ 0.05). LSD: the least significant difference. *** means highly significant. SA^#^ = (Specific activity), RA^##^% = relative activity percentage = $\frac{{{\text{treatment}} - {\text{control}}}}{{{\mathrm{control}}}} \times 100$. ^##^C% = change percentage = $\frac{{{\text{treatment}} - {\text{control}}}}{{{\mathrm{control}}}} \times 100$

The activities of glutamic pyruvic transaminase (GPT) and glutamic oxaloacetic transaminase (GOT) were measured in adult honeybee workers treated with various plant extracts (0.1%, w/v) for 48 h (Table 6). The experimental groups showed statistically significant differences in GPT and GOT activities compared to the control. Specifically, GPT activity was increased in all treatments by the 3^rd^ day, except for *E. guineensis* extract 2, that significantly decreased by 26.42%. The GPT of honeybees was increased in response to *A. nilotica* extracts (3970.95 mU/g. b. wt.), followed by *E. guineensis* extract1 (3639.4 mU/g.b.wt.), and *C. roseus* (3478.1 mU/g.b. wt.). The percentage increase in GPT activities in honeybees in response to treatment with extracts of *A. nilotica*, *E. guineensis* extract 1 and *C. roseus* were 16.3%, 6.6%, and 1.9%, respectively.

**Table 6:** Effect of plant extracts on infected bees, Total soluble proteins, AChE, ALP and GST on the 5- day and 9-day post-treatment:

Extract treatment/ day post-infection	Total soluble proteins (mg protein./g body weight)				AChE µg AchBr /min/g. body weight				ALP (U/g. b. wt.)				GST			
	5-dpt		9-dpt		5-dpi		9-dpi		5-dpt		9-dpt		5-dpt		9-dpt	
	Con	RC	Conc	RC	SA	RA	SA	RA	SA	RA	SA	RA	SA	RA	SA	RA
*Acacia nilotica* extract	40.53 ± 1.76	44.9	35.033^cd^	− 23.22	136.67 ± 7.57^b^	28.12	152.3 ± 11.015 ^b^	54.92	1273.33 ± 87.37 ^c^	55.85	840.33 ± 53.48 ^c^	− 16.52	45 ± 2.65 a	− 8.94	37.67 ± 3.79 c	− 32.7
*Elaeis guineensis* extract1	46.2 ± 4.42	65.2	31.867^d^	− 30.16	151.67 ± 3.78 ^ab^	42.19	182.33 ± 6.81 ^b^	85.43	3206.67 ± 310.05 ^a^	292.49	1140 ± 9.54 ^a^	13.24	24.33 ± 3.21 b	21.42	38 ± 2.65 c	− 32.1
*Elaeis guineensis* extract2	52 ± 4.45	85.9	36.97 ^c^	− 18.99	165 ± 4.36 ^a^	54.68	325.67 ± 36.14 ^a^	231.20	2163.67 ± 176.01 ^b^	164.83	958.33 ± 53.46 ^b^	− 4.80	29.67 ± 2.52 b	110.66	56 ± 1 a	0.00
*Catharanthus roseus* extract	47.6 ± 3.41	70.2	50.7 a	11.103	50.33 ± 4.51 ^d^	− 52.82	52 ± 2 ^c^	− 47.12	535 ± 57.66 ^d^	− 34.52	1214.33 ± 35.02^a^	20.63	42 ± 2 a	20.63	47 ± 3.61 b	− 16.0
Untreated infected control	*27.9* ±	0	45.633b	0	106.67 ± 9.07 ^c^	0	98.33 ± 9.02 ^c^	0	817 ± 37.51 ^d^	0	1006.67 ± 40.41^b^	0	45.67 ± 3.05 a	0	56 ± 3.61 a	0
LSD	32		3.32		11.31		32.12		303.85		75.75		75.75		5.65	
P _0.05_			> .0001***		> .0001***		> .0001***		> .0001***		> .0001***		> .0001***		> .0001***	

Values given are the mean ± SD.

Means, within column, bearing different letters are significantly different (ONE Way ANOVA, Tukey’s HSD test, p ≤ 0.05). LSD: the least significant difference. *** means highly significant. ^#^ SA = (Specific activity). ^#^ Conc. = concentration.

^##^RA% = relative activity percentage = $\frac{{{\text{treatment}} - {\text{control}}}}{{{\mathrm{control}}}} \times 100$. ^##^C% = change percentage = $\frac{{{\text{treatment}} - {\text{control}}}}{{{\mathrm{control}}}} \times 100$.

The activity of GOT was decreased by the 3rd day of treatment compared to control except for *E. guineensis* 2 which was significantly increased by 60.5%. The extracts of *A. nilotica*, *E. guineensis* 1 and *C. roseus* strongly reduce the GOT activity to be (67.03, 59.82, and 39.78, U/g. b.wt., respectively), compared to control honeybees. The activity of AChE was assessed in honeybees in response to the plant extracts, the extracts of *A. nilotica* had the mild effect on the biology of the bees, with about 9.7% reduction compared to the control bees (123.3 µg AchBr /min/g body weight). The extracts of *E. guineensis* extract 1, 2, and *C. roseus* significantly increase the AChE activity by 261.0, 194.3, and 67.3%, respectively, compared to the control. The activity of ALP in the honeybees in response to the different plant extracts was assessed. The extracts of *E. guineensis* 1 and 2 had the highest inhibitory effect on the activity of ALP, compared to uninfected control of honeybees. The specific activity of ALP in honeybees in response to *E. guineensis* 1 and 2 was significant decreased into 376.6, 347.4 μg phenol/ g body weight, compared to the untreated uninfected control (1293.3 μg phenol/ g body weight). However, the extracts of *A. nilotica* and *C. roseus* had a slight effect on the induction of ALP activity (784.0 and 603.2 μg phenol/g body weight), compared to the ALP activity of control honeybees (1293 μg phenol/ g body weight). The activity of GST in the honeybees in response to the plant extracts was determined as shown in Table 4. The extracts of *E. guineensis* 1 and 2 strongly suppress the activity of GST by − 46.7 and − 35.0%, respectively, compared to the uninfected and untreated bees (45.67 ± 3.05 ^a^ m mole substrate conjugated /min/g. b. wt), with *p* values < 0.001. Thus, from the biochemical analyses of the uninfected healthy honeybee, *A. nilotica* had the lowest toxicity levels in the biology of the bees that was relatively similar to untreated honeybees.

### Biochemical of honeybee artificially infected with *Nosema* in response to the plant extracts

**Fig. 3:**
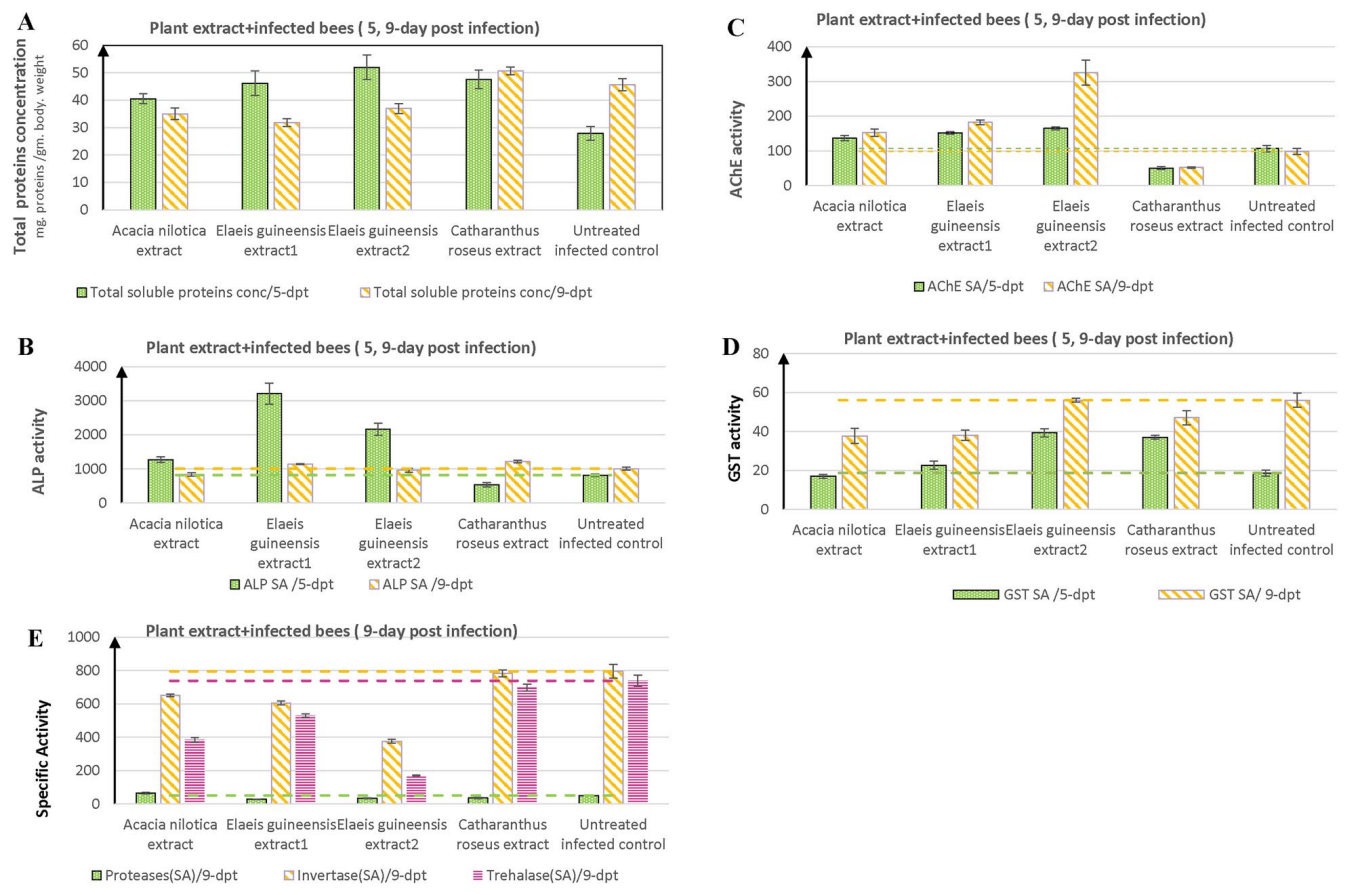
Biochemical properties of the honeybees infected with *Nosema* spores in response to the different plant extracts after 5 days and 9 days of post infection. The estimated biochemical parameters were total protein contents (**A**), ALP activity (**B**), Acetylcholine esterase (**C**), GST activity (**D**) and protease, invertase and trehalase activities (**E**).

The effect of the plant extracts at 0.1%, on the biochemical parameters of honeybees infected with *Nosema* species was determined after 5 and 9 day post-treatment. The different biochemical properties of the honeybees including total protein, and the activities of AchE, ALP, and GST are illustrated in Table 6. A significant increase in total proteins of bees in response to the plant extracts, by the 5^th^ day of post-infection, followed by an obvious decrease by the 9^th^ days of post-infection (Fig. 3). The biomarker of neurotoxicity (AChE) was determined on the 5 day and 9 day post-treatment, in response to the treatment with the plant extracts. By the 5^th^ days of post-infection, the extracts of *A. nilotica*, *E. guineensis* 1, and 2 increases the activity of AChE of the *Nosema* infected honeybee by about 28.12, 42.19, and 54.6%, respectively. The activity of AChE in infected bees was reduced by about 52.8, in response to the extracts of *C. roseus.* As well as, on the 9^th^ day post-infection, the plant extracts showed an increase in the AChE activity, unlike the obvious decrease by *C. roseus*, by about 47.12%, compared to the control.

The biomarkers of metabolic alteration, detoxification, and defense-related enzymes such as ALP, and GST were determined in the infected honeybee in response to the plant extracts. By the 5^th^ day post-infection, the ALP activity was increased significantly in response to extracts of *A. nilotica*, *E. guineensis* 1 and 2, with about 55.8, 292.4, and 164.8%, respectively, compared to the untreated control (Table 6). The extracts of *C. roseus* gave about 34.52% reduction on the activity of ALP levels on the 5^th^ day post-infection. However, at 9^th^ days post-infection, the ALP activity was decreased compared to that of 5 d.p.i, by about 16.5, 13.2, 4.8, and 20.6% for treatment with *A. nilotica*, *E. guineensis* extract 1, 2 and *C. roseus*, compared to the respective controls. As well as the GST activity of the infected honeybees was determined in response to the plant extracts treatment (Table 6). The tested plant extract showed a mild increase in the activity of GST, *A. nilotica* reduces the GST activity by about 8.9%, compared to the control.

The proteolytic activity and carbohydrate hydrolyzing enzymes of the infected honeybees were determined in response to various plant extracts. The protease activity of the infected honeybees was increased by *A. nilotica* extracts to 65.6 μg/min/g, compared to the untreated control (50.33 μg/min/g. b. wt) (Table 7). As well as, the activity of protease in honeybee infected with *Nosema* spores in response to the extracts of *E. guineensis* 1, 2 and *C. roseus* was significantly reduced by about 43.3, 32.6 and 29.8%, respectively, compared to the untreated infected honeybees as control. The enzymatic activities of the carbohydrate hydrolyzing enzymes, namely invertase and trehalase were assessed as a biochemical indicator of the honeybee’s utilization efficiency of the ingested sugars, at 9 days post infection. The activity of invertase and trehalase in the honeybee infected with *Nosema*, was reduced by about 18.1%, 47.9%, respectively, in response to *A. nilotica* extracts, however, the highest deleterious effect on these enzyme activities was shown in response to methanolic extract of *E. guineensis* 2, compared to control. The extract of *C. roseus* had a mild effect on invertase and trehalase activity of honeybees, as revealed from their relative activities (~ 1–5%), compared to the untreated controls. Thus, from the toxicity assessment of the plant extracts to the uninfected healthy honeybees, selective targetability to the *Nosema* infecting the bees, biochemical and physiological properties of the bees, the methanolic extract of *A. nilotica* had the most affordable anti-nosemosis activity and less toxicity to the honeybees. So, the methanolic extract of *A. nilotica* has been further chemical analyzed to elucidate the most active constituents.

**Table 7:** Effect of plant extracts on infected bees, proteolytic activity, and carbohydrate hydrolyzing enzymes.

Extract treatment (9-day post-treatment)	Proteases (μg alanine/min/g. b. wt.)		Invertase		Trehalase	
	SA	RA%	SA	RA%	SA	RA%
*Acacia nilotica* extract	65.67 ^a^ ± 5.46	30.463	650.33^b^ ± 6.80	− 18.09	384.33^c^ ± 13.79	− 47.95
*Elaeis guineensis* extract1	28.53 ^c^ ± 1.28	− 43.31	605.33^b^ ± 11.93	− 23.76	529.33^b^ ± 10.06	− 28.31
*Elaeis guineensis* extract2	33.9 ^c^ ± 2.98	− 32.6	375.67^c^ ± 12.50	− 52.69	171.33 ^d^ ± 3.21	− 76.79
*Catharanthus roseus*	35.33^c^ ± 3.617	− 29.80	782 ^a^ ± 20.07	− 1.51	697.33^a^ ± 20.98	− 5.55
Untreated infected control	50.33^b^ ± 4.01	0	794 ^a^ ± 41.32	0	738.33^a^ ± 33.29	0
LSD	6.786		40.32		35	
P _0.05_	> .0001***		> .0001***		> .0001***	

Values given are the mean ± SD.

Means, within column, bearing different letters are significantly different (ONE Way ANOVA, Tukey’s HSD test, p ≤ 0.05). LSD: the least significant difference. *** means highly significant. ^#^SA = (Specific activity).

^##^RA% = relative activity percentage = $\frac{{{\text{treatment}} - {\text{control}}}}{{{\mathrm{control}}}} \times 100$.

### GC–MS/MS analysis of *A. nilotica* methanolic crude extract:

**Fig. 4:**
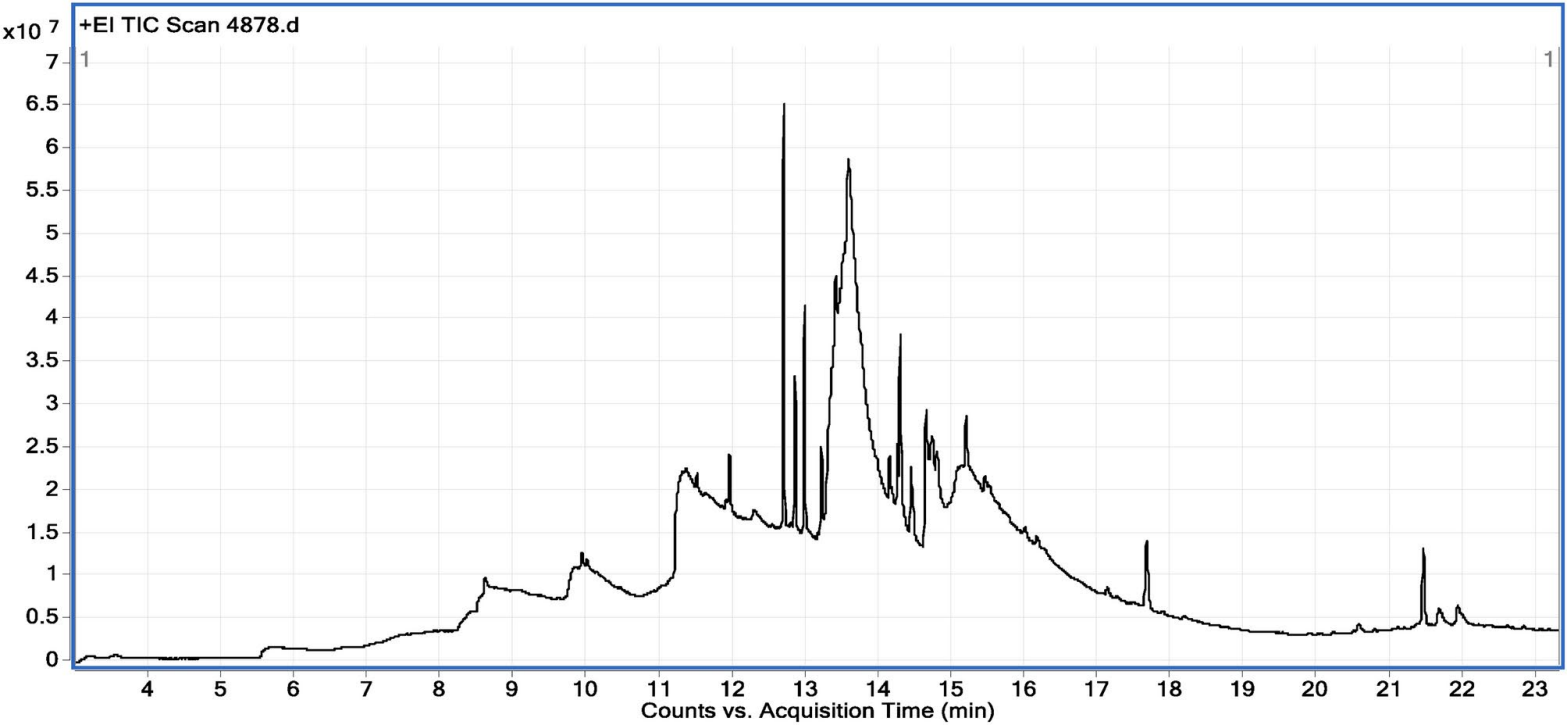
Total ion current chromatograms (Counts vs. Acquisition Time (min)) of the LC–MS analysis of *A. nilotica* extracts.

The methanolic extract of *A. nilotica* showing the greatest reduction in spore load was further subjected to GC–MS analysis. The *A. nilotica* methanolic extract was selected for its affordable activity against *N. ceranae* and *N. apis* and safety for bees at the tested concentrations. The chemical constituent of the crude extract of *A. nilotica* was determined, as revealed from the total ion chromatogram (Fig. 4). Twenty-one chemical compounds were identified, with their concentration, retention times, molecular weights, and molecular formula, as listed in Table 8. From the GC–MS metabolic profiling (Table 8), the methanolic extract of *A. nilotica* had a major eight compounds as myristic acid (C_14_H_28_O_2_), 2,4,6-trihydroxybenzoic acid, 1,2-dihydroxy-benzene, 2-butyloctanoic acid, phytol, butein, oleic acid, and rhamnetin. Myristic acid has been reported with the highest peak area 48.17%, compared to the total compounds, this compound has been known for its powerful antimicrobial properties. The percentage of the compounds 2,4,6-trihydroxybenzoic acid, 1,2-dihydroxybenzene (C_6_H_6_O_2_), 2-butyloctanoic acid, phytol, butein, oleic acid, and rhamnetin were 13.6, 10.9, 7.5, 4.3, 4.0, 4.0, 2.6 and 2.1%, respectively. From the total ion chromatogram, myristic acid was resolved at 13.6 min, 2,4,6-Trihydroxybenzoic acid at 11.3 min, 1,2-dihydroxybenzene at 10.03 min, 2-Butyloctanoic acid (C_12_H_24_O_2_) was at 8.6 min, and phytol (Acyclic diterpene) (C_20_H_40_O) at 12.6 min. The presence of saturated long-chain fatty acids such as myristic acid, oleic acid, linoleic acid, and arachidic acid refers believed to enhance the biological functions of bees, by providing fats, generating energy, with plausible antimicrobial properties. The potential anti-nosemosis effect may result from the release of these lipids onto the surface of microbial cells, thus impeding spore germination and microbial growth.

**Table 8:** Common name and chemical structure of *Acacia nilotica* methanolic crude extract isolated components by GC.

No	RT	Compound name		Molecular formula (Molecular weight (g/mol))	Area sum%
1	8.6	2-Butyloctanoic acid	200.32	C_12_H_24_O_2_	4.38
2	10.03	1,2-dihydroxybenzene	110.11	C6H6O2	7.57
3	11.3	2,4,6-Trihydroxybenzoic acid	170.12	C_7_H_6_O_5_	10.9
4	11.95	3-O-Methylgallic acid	184.15	C_8_H_8_O_5_	0.76
5	12.3	4'-Hydroxy-7-methoxyflavone	268.26	C_16_H_12_O_4_	1.87
6	12.6	**Phytol**	296.5	C_20_H_40_O	4.01
7	12.8	Linoleic Acid	280.4	C_18_H_32_O_2_	1.4
8	12.98	Rhamnetin	316.26	C_16_H_12_O_7_	2.19
9	13.6	Myristic acid	228.37	C_14_H_28_O_2_	48.17
10	14.13	Geranyl Isovalerate	238.37	C_15_H_26_O_2_	0.89
11	14.3	**Phytol**	296.5	C_20_H_40_O	2.18
12	14.4	Arachidic Acid	312.5	C_20_H_40_O_2_	1.09
13	14.6	Oleic Acid	282.5	C_18_H_34_O_2_	2.6
14	14.7	13,16-Docosadienoic acid	336.6	C_22_H_40_O_2_	1.96
15	14.8	trans-Trimethoxyresveratrol	270.32	C_17_H_18_O_3_	1.4
16	15.19	Butein	272.25	C_15_H_12_O_5_	4
17	16.15	3-Methoxy-4-hydroxyphenylglycol	184.19	C_9_H_12_O_4_	0.58
18	17.6	scopoletin	192.17	C_10_H_8_O_4_	1.3
19	21.43	dihydroquercetin	304.25	C_15_H_12_O_7_	1.38
20	21.6	Syringaresinol	418.4	C_22_H_26_O_8_	0.54
21	21.95	15-Octadecenal	266.5	C_18_H_34_O	0.81

The existence of such beneficial components in significant proportions, along with other phytochemicals (e.g., phytol, diterpenoid, and carboxylic ester such as Geranyl Isovalerate with insecticidal properties), which could pose risks to bees in small quantities, elucidates the safety of these extracts when used at appropriate concentrations, that could target the germination of *Nosema* spores in the midgut of honeybees. Certain phytochemicals such as oleic acid, palmitic acid, and linolenic acid, were known for their potential inhibitory effect on the spore forming microorganisms, as detected in minimal quantities in *A. nilotica* extracts.

### Molecular modeling analysis

**Fig. 5:**
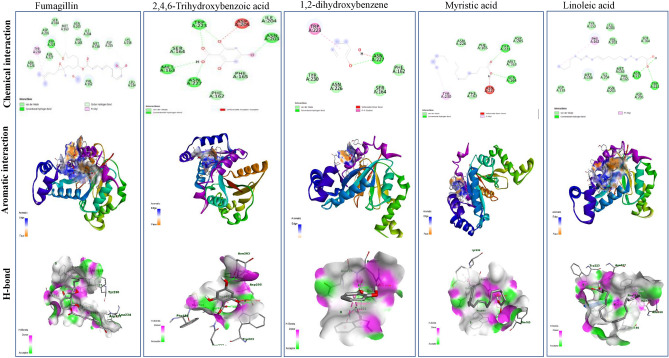
The molecular docking analysis of the most frequent compounds of *A. nilotica* namely 2,4,6-Trihydroxybenzoic acid, 1,2-dihydroxybenzene, myristic acid and linoleic acid with the ATP binding protein of *N. ceranae,* compared to Fumagillin as reference compound. The 2D chemical interaction, aromatic interaction and H-bonds of the legend and the ATP binding protein of *N. ceranae* were shown.

**Fig. 6:**
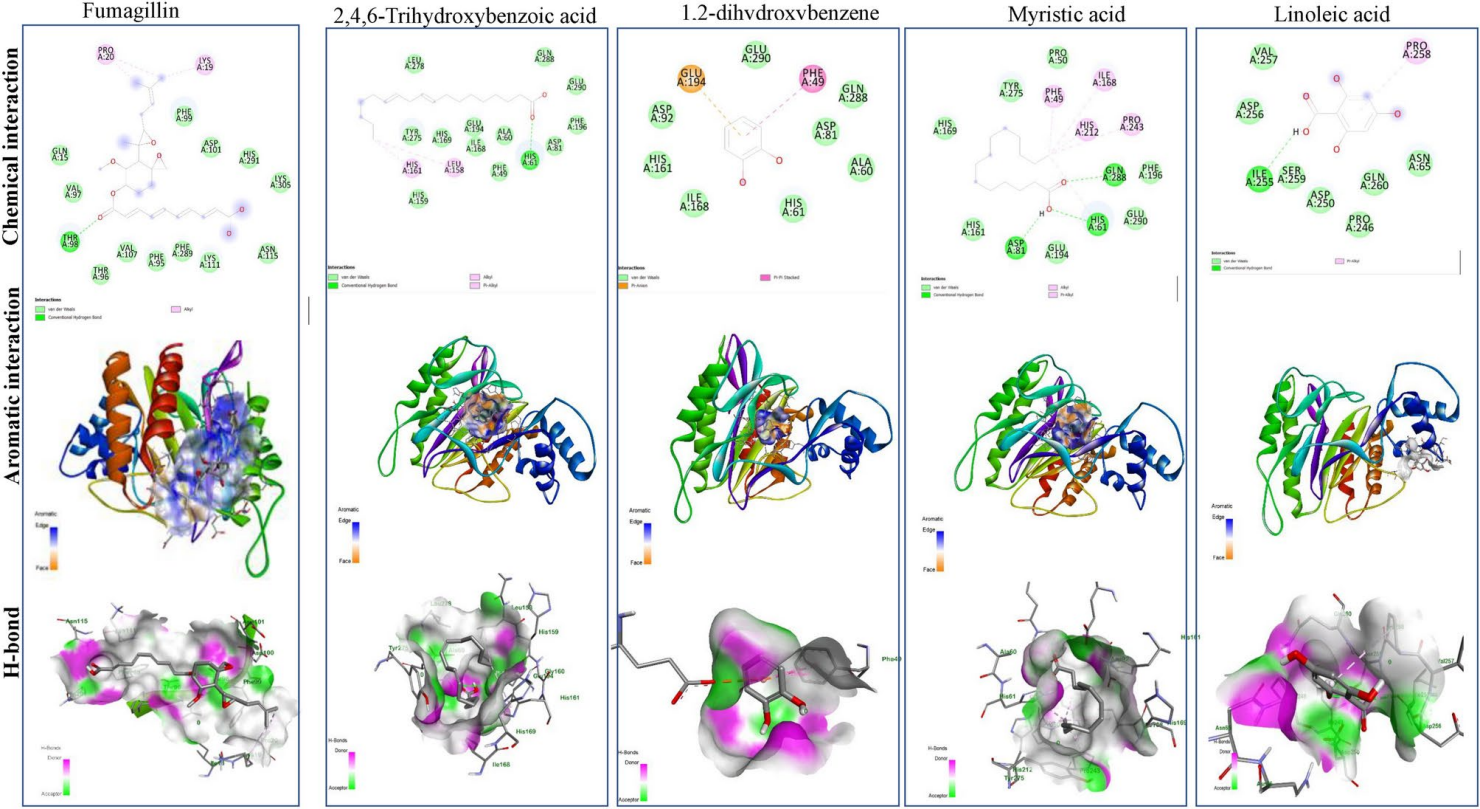
The molecular docking analysis of the most frequent compounds of *A. nilotica* namely 2,4,6-Trihydroxybenzoic acid, 1,2-dihydroxybenzene, myristic acid and linoleic acid with the Methionine aminopeptidase of *N. ceranae* compared to Fumagillin, as reference compound*.* The 2D chemical interaction, aromatic interaction and H-bonds of the legend and the ATP binding protein of *N. ceranae* were shown.

**Fig. 7:**
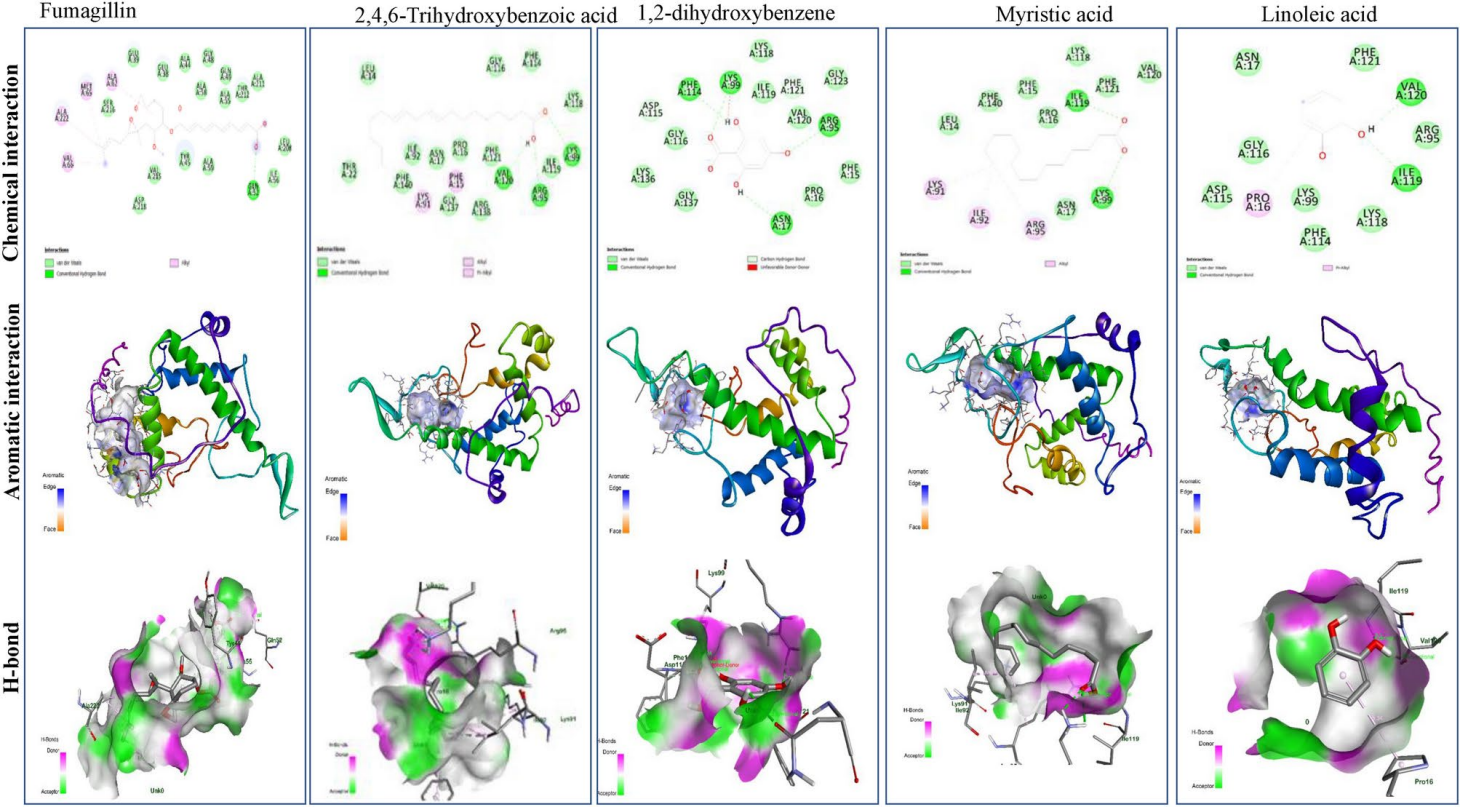
The molecular docking analysis of the most frequent compounds of *A. nilotica* namely 2,4,6-Trihydroxybenzoic acid, 1,2-dihydroxybenzene, myristic acid and linoleic acid with the polar tube protein 2 of *N. ceranae,* compared to Fumagillin as reference compound*.* The 2D chemical interaction, aromatic interaction and H-bonds of the legend and the ATP binding protein of *N. ceranae* were shown.

The docking analysis of the most frequent components of *A. nilotica* extracts namely 2,4,6-trihydroxybenzoic acid, 1,2-dihydroxybenzene, myristic acid, linoleic acid, were conducted to illustrate their binding affinity to the target enzymes of *Nosema* species using fumagillin as reference compound. Several enzymes such as ATP binding protein, methionine aminopeptidase, and polar tube protein 2 in *Nosema ceranae* were considered*.* The binding affinities of the docked complexes, and the hydrophobic residues, H-bonds, and distances of each docked complex were summarized in Table 9. The tested compounds of *A. nilotica* had an affordable binding affinity with the ATP binding protein of *Nosema* sp, as summarized from the distance of binding, energy of binding affinity, and number of hydrophobic interactions. The highest binding affinity with the ATP binding proteins of *Nosema* was reported for 2,4,6-trihydroxybenzoic acid, followed by 1,2-dihydroxybenzene, myristic acid, and linoleic acid, that were − 5.2, − 4.5, − 4.4 and − 4.3 kcal/mol, respectively, compared to fumagilin (− 7.4 kcal/mol). So, the binding affinity of 2,4,6-trihydroxy-benzoic acid was slightly lower than fumagilin by about 30%, ensuring the partial efficiency of the tested compounds. While the binding affinity of 1,2-dihydroxybenzene, myristic acid, and linoleic acid was lower than fumagilin by about 40%, as revealed from the binding energies with ATP binding protein of *Nosema* sp. The 2D and 3D of the ATP binding protein in *Nosema* was illustrated in Fig. 5. The fumagillin had one conventional hydrogen bond and two carbon-hydrogen bonds in the amino acids’ residues TRP223, MET 163, and ASP 235. The 2,4,6-trihydroxybenzoic acid had five conventional hydrogen bonds in the residues ASN 203, TRP 223, MET 163, and ASN 227, while 1,2-dihydroxybenzene had one conventional hydrogen bond in the residues ASN 227. Myristic acid has two conventional hydrogen bonds in the amino acids’ residues TRP 223, and SER 164. Linoleic acid has two conventional hydrogen bonds in the amino acids residues ASN 227 and TRP 223. The 2D and 3D of methionine aminopeptidase 2 of *N. apis* was illustrated in Fig. 5. From the in silico studies, the binding affinity of 2,4,6-trihydroxy-benzoic, 1,2-dihydroxybenzene, and myristic acid methionine aminopeptidase of *N. apis* was − 5.1 kcal/mol, while linoleic acid had − 5.6 kcal/mol. From the 2D chemical interactions, linoleic acid shows one conventional hydrogen bond in the amino acid’s residue HIS A6 (Fig. 6, Table 9). Myristic Acid shows three conventional hydrogen bonds in the amino acids’ residues GLN 288, HIS 61, and ASP 81, while the 2,4,6-Trihydroxybenzoic acid shows one conventional hydrogen bond in the amino acids’ residue ILE 255. Fumagillin had one conventional hydrogen bond in the amino acid residue THR 98. The aromatic interaction showing the region of the interaction of methionine aminopeptidase 2 *N. apis* with fumagillin, linoleic Acid, 1,2-dihydroxybenzene, myristic acid, and 2,4,6-Trihydroxybenzoic acid. The H-bonds interaction as donors (pink color) and acceptors (green color) showing the region of methionine aminopeptidase 2 *N. apis* with fumagillin, linoleic acid, 1,2-dihydroxybenzene, myristic acid, and 2,4,6-Trihydroxybenzoic acid. The 2D and 3D of the polar tube protein 2 in *V. ceranae* was illustrated in Fig. 7. From the binding affinities of the compounds 2,4,6-trihydroxy-benzoic acid, linoleic acid, myristic acid and 1,2-dihydroxybenzene were -6.8, -6.9, -6.3, and -5.4 kcal/mol, respectively, compared to fumagillin (-8.8 kcal/mol), with the polar tube protein 2 of *V. ceranae*. Linoleic Acid had three conventional hydrogen bonds in the amino acid residues LYS99, ARG95, and VAL120. The compound 2,4,6-trihydroxybenzoic acid had four conventional hydrogen bonds and two carbon hydrogen bonds in the residues ARG95, ASN17, LYS99, PHE114, VAL120 and ASP115. Myristic acid had two conventional hydrogen bonds in the amino acid residues LYS A: 99 and ILE A: 119. The compound 1,2-dihydroxybenzene had two conventional hydrogen bonds in the amino acids residues VAL120 and ILE119, while, fumagillin shows one conventional hydrogen bond in the amino acid residue GLN52.

**Table 9:** The binding affinities, hydrophobic residues, H-bonds, and distances of each docked complex.

Protein	Ligand	Hydrophobic residues	Distance	H-bond residues	Distance	Binding Affinity (kcal/mol)
ATP binding protein in *Vairimorpha ceranae*	Fumagillin	TRP 223 TYR 230	4.31444 4.75903	TRP 223 ASP 235 MET 163	2.44932 3.41536 3.41784	− 7.4
	2,4,6-Trihydroxybenzoic acid	–	–	ASN 203 TRP 223 TRP 223 ASN 227 MET 163	2.40565 2.92903 2.25775 2.10923 2.61371	− 5.2
	1,2-dihydroxybenzene (Catechol)	TRP 223 TRP 223	3.89763 3.89802	ASN 227	2.81157	− 4.5
	Myristic Acid	TYR 230	4.57898	SER 164 TRP 223	2.66801 2.61936	− 4.4
	Linoleic Acid	PHE 162	4.15817	TRP 223 ASN 227	2.83308 2.35589	− 4.3
Methionine aminopeptidase *2_Nosema apis*	Fumagillin	PRO20 LYS19 PRO20	4.27609 4.21396 4.37149	THR98	2.27808	− 7.1
	2,4,6-Trihydroxy-benzoic acid	PRO258	5.05812	ILE255	2.87229	− 5.1
	1,2-dihydroxybenzene	PHE49	4.39579	–	–	− 5.1
	Myristic Acid	ILE168 PRO243 PHE49 HIS61 HIS212	4.69276 4.35848 4.21076 4.98854 4.77046	HIS61 GLN288 ASP81	2.63082 2.73962 2.10402	− 5.1
	Linoleic Acid	LEU158 HIS161	4.9267 4.57288	HIS61	2.5391	− 5.6
Methionine aminopeptidase 2 *Apis mellifera*	Fumagillin	ARG367 PRO442 ARG367 LEU324 LEU324 HIS227 PHE362 PHE362 TYR440 TYR450 TYR450	5.12679 4.99218 4.38649 5.09874 4.40851 3.79471 4.81708 4.92566 5.11487 4.42659 4.06007	GLN453PRO441	2.64342 2.57549	− 8.8
	2,4,6-Trihydroxy-benzoic acid	ALA336 LYS338	4.86179 4.68915	ILE329 ARG152 ARG156	2.66499 2.48513 3.72383	− 6.2
	1,2-dihydroxybenzene	ILE256 ILE334	4.77092 5.37418	GLY249	2.29128	− 5.1
	Myristic Acid	ALA35 LYS38 LYS39 LYS47	3.73569 4.713 4.42678 3.65739	ALA53	2.59147	− 5.8
	Linoleic Acid	ILE334	4.91247	ASP247	2.35077	− 6.1
Polar tube protein 2_Vairimorpha ceranae	Fumagillin	ALA55 ALA55 ALA58 ALA59 ALA59 ALA62 ALA62 VAL66 ALA222 ALA222 MET65 VAL66 TYR45 TYR45	4.68413 3.73252 4.27331 4.59317 4.79799 5.04707 4.90297 4.33409 3.94927 3.69458 4.72876 4.24912 5.33575 5.32301	GLN52	2.49671	− 8.8
	2,4,6-Trihydroxy-benzoic acid			ARG95 LYS99	2.59285 2.24261 2.68787 2.30282 3.45625 3.2437	− 6.8
	1,2-dihydroxybenzene	PRO16	5.33642	ILE119 VAL120	2.21578 1.91926	− 5.4
	Myristic Acid	LYS91 ILE92 ARG95	3.74049 4.38289 4.33203	LYS99 LYS99 ILE119	2.75972 2.96119 2.21883	− 6.3
	Linoleic Acid	PRO16 LYS91 ILE92 ILE92 LEU14 ILE92 LYS91 PHE140	4.94256 4.80095 4.3693 5.1502 4.48048 5.32714 4.48663 4.77857	ARG95 LYS99 LYS99 VAL120	2.79441 3.09932 2.73508 2.09622	− 6.9

## Discussion

Honeybees are one of the most economically important insect for human nutrition and medications, however, these bees are vulnerable to various biotic stressors, causing a significant colony loss, mortality, rapid depopulation^1^. Nosemosis is an intestinal disease of the adult honey bee, caused by the indigenous fungal microsporidia pathogen *Nosema apis*, *N. ceranae* and *N. neumanii*^2–5^.

So, this study was to scrutinize plant extracts with higher selectivity to target the spores of *Nosema* with little/ nil toxicity to healthy honeybees. Several plants were selected based on their ethnological and pharmaceutical properties namely *Acacia nilotica, Elaeis guineensis, and Catharanthus roseus*^38,39,41,44,45,47,49^. The methanolic extracts of the tested plants were initially assessed for their cytotoxicity to the natural, uninfected honeybees. The extract of *A. nilotica* had the lowest toxicity to the honeybee, while *C. roseus* and *Elaeis guineensis* causing an abnormal behavior, shrinkage and dryness, and reduced flight ability. So, the concentration of 0.1% was chosen for further antimicrobial screening as minimal toxicity for bees. Similar results ensuring the mild toxicity of *A. nilotica* on the animals, and their potential antibacterial properties^38,39,76^. The higher cytotoxicity of *Catharanthus roseus* to the honeybees was ensured frequently, that might be due to their toxic alkaloids such as vinblastine and vincristine, that disrupting the nervous system of the insects, and cell division of the plant^77–79^. *Nosema* infected honeybees have lower trehalose sugar levels in their hemolymph, that might have a negative effect on the flight^34,80,81^. Similar studies report the cytotoxic activity of plant extracts to the bees^27^. Essential oils like thymol and oxalic acid vary in toxicity levels^82^. *Acacia nilotica* and *Catharanthus roseus* extracts exhibited promising anti-microsporidian properties leading to reduced spore counts compared to the control group and other extracts with a reduction by about 37.84 and 27.03%, at 5^th^ day post-infection. *Acacia nilotica* was most effective against *Nosema* infection at 0.1% with minimal toxicity, while *C. roseus* caused a significant mortality rate. Similarly, the probiotics of bees were found to reduce the *Nosema* species spore counts and increased the colony strength, with obvious higher mortality rate^83^. *Elaeis guineensis* extracts displayed a minimal reduction to the spores load per bee at the tested concentration. They may require further investigation with different concentrations or extraction methods. The antimicrobial properties of medicinal plant extracts and the silver nanoparticles displayed a strong activity against honeybee pathogens^84^. Propolis consumption reduces the *N. ceranae* infection in honeybees^33^. El-Seedi et al.^14^ reported the efficacy of the extracts *L. nobilis* in controlling *Nosema ceranae* infections in honeybees. Similarly, the extracts of *Agaricus blazei* had a positive effect against *Nosema* species^85^. As well as, the herbal plant extract had a significant activity against *N. ceranae* infection and positively influenced the honey bee colony strength and health. Ptaszyńska and Załuski^31^ reported that *E. senticosus* extract has shown promising results in combating nosemosis in honeybees. It effectively reduced the level of nosemosis both as a treatment after infection and as a preventive measure. The extract’s adaptogenic properties are believed to enhance honeybee immunity, as indicated by increased levels of phenoloxidase in the hemolymph of treated bees. The microscopic results of *Nosema* species infecting the honeybees in response to the plant extracts were confirmed from the qPCR analysis. The concentration of *Nosema* (*Vairimorpha*) spores in the in response to the plant extract were assessed after 5 and 9-days post-infection in bioassay was determined by Real-Time PCRassay depending on 2^-ΔΔ*CT*^ method in analyzing fungal load of the samples qPCR analysis confirmed the effectiveness of *Acacia nilotica* and *Catharanthus roseus* extracts in reducing Nosema spore load observed microscopically. The qPCR analysis revealed the significant variation in the spore load of *N. apis* and *N. ceranae* between the treated and untreated positive controls. Several studies reporting the sensitivity of qPCR to distinguish of *N. apis*, *N. ceranae* and *N. bombi* ^86–88^. So, from the microscopic and qPCR analyses, a significant reduction to the load of spores of *N. apis* and *N. ceranae*” was observed at 5 and 9 days post infection in response to *A. nilotica* and *C. roseus* methanolic extracts. So, this is first report describing the anti-microsporidian activity of *A. nilotica* and *C. roseus* in combating Nosema infections in honeybee populations, with mild toxicity effect to the honeybee viability. Similarly, the extract of *A. nilotica* had an efficient antimicrobial. As well as, the insecticidal, fungicidal and antibacterial activity of *A. nilotica* pods extracts was reported^40,76,89^. As well as, the biological activity of *C. roseus* towards various pathogenic bacteria and fungi were reported, that might be due the presence of vinblastine and vincristine alkaloids^90^.

The biochemical and physiological responses of uninfected and infected adult honey bees due to the plant extracts, provide valuable insights on the effect of these plant extracts on the bee health^91^. The extract of *A. nilotica* increases the total protein content of the honeybees, suggesting potential nutritional benefits, similar results were reported the increasing on the protein contents of honeybees in response to the natural products, decreasing the honeybee colony losses during winter^92^. The insects implement the metabolic resistance to biotic stressors by activating the cytochrome P450 monooxygenases, glutathione transferases, and carboxylesterases^93,94^. The antioxidant activity of flavonoids is attributed to their ability to scavenge reactive oxygen species and inhibit oxidative enzymes, as a promising candidate for disease treatment^95^. The toxicity of the plant extracts was revealed from the activities of the enzymatic biochemical markers GPT, GOT, AchE, ALP, and GST. The GPT activity was increased in all treatments by the 3rd day, the GPT activity of honeybees was increased in response to *A. nilotica* extracts, followed by *E. guineensis* extract 1, and *C. roseus*. The GOT activity was decreased by the 3rd day of treatment compared to control except for *E. guineensis* 2. The extracts of *A. nilotica*, *E. guineensis* 1 and *C. roseus* strongly reduce the GOT activity, compared to control honeybees. The activity of AChE was assessed in honeybees in response to the plant extracts, *A. nilotica* extract had the mild effect on the biology of bees, with about 9.7% reduction compared to the control bees (123.3 µg AchBr /min/g body weight). Increased the GPT activity reveals the higher amino acid metabolism, suggesting the plant extracts stimulate protein synthesis or breakdown for energy production. The activity of ALP in honeybees in response to *E. guineensis* 1 and 2 was significantly decreased compared to the untreated uninfected control. The extracts of *A. nilotica* and *C. roseus* had a slight effect on the induction of ALP activity, compared to the control honeybees. The extracts of *E. guineensis* 1 and 2 strongly suppress the activity of GST. So, from the biochemical analyses, *A. nilotica* had the lowest toxicity levels to the biology of the bees. A significant increase in total proteins of bees in response to the plant extracts, by the 5th day of post-infection, followed by an obvious decrease by the 9^th^ days of post-infection. The extracts of *A. nilotica* increase the activity of AChE of the *Nosema* infected honeybee by about 28.12%. The ALP activity was increased significantly by extracts of *A. nilotica* by 55.8, compared to the untreated control. The extracts of *C. roseus* reduced the activity of ALP by about 34.52% on the 5th day post-infection. The protease activity of the infected honeybees was significantly increased by *A. nilotica* extracts, compared to the untreated control. The activity of invertase and trehalase in the honeybee infected with *Nosema* was reduced by about 18.1, 47.9%, respectively, in response to *A. nilotica* extracts. The lack of protein digestion reduces the protein contents of the hypopharyngeal glands, which is crucial for producing food by the nurse bees that is used to feed developing honey bees^96,97^. The ALT and AST are crucial amino acid metabolizing enzymes, a link between carbohydrate and protein metabolism, and their higher activities points to the tissue damage in humans due to the bacterial infections. AChE is a crucial enzyme in bees nerves functionality, facilitates the nerve impulse transmission and acetylcholine hydrolysis^98–102^. The change in AChE activity was observed in *A. mellifera* bees during winter, indicating stress^103^. The exposure to deltamethrin insecticide leads to a decrease in AChE activity in honey bees^100^. Glutathione S-transferase (GST) plays a crucial role in detoxifying the xenobiotics in honey bees, with the highest activity in the midgut^104^. The activity of GSTs can be influenced by diet, with the highest activity observed in bees fed of 30% sugar solution^105,106^. GST is a Phase II enzyme, detoxifying the toxic compounds by catalyzing the conjugation of reduced glutathione^99,101,107^. The total proteins were generally increased at 5 days post-infection and decreased after 9 days, suggesting complex interactions between *Nosema* infection, extract treatment, and protein metabolism. The plant extracts showed an increased protein levels on 5th day, followed by strong decrease on the 9th day. The initial increasing on the protein contents could be due to the bee’s immune response to *Nosema* infection by the extracts, the later decrease in protein levels could be due to the increased protein breakdown for energy production as the bees fight the infection. The *Nosema* spores proliferate within the cells of the gut lining of adult bees, thereby decreasing host intestine function and interfering with nutrient digestion and uptake. The loss of nutrient uptake capacity due to *Nosema* infection may also lead to a decrease in energy reserves and reduction in longevity and activity of the host. By the 9^th^ d.p.i, the GST activity was decreased in all treatment groups compared to 5^th^ day. *Acacia nilotica* didn’t significantly affect the GST activity on 5^th^ day, suggesting it might not induce the same detoxification pathways as the other extracts. This study reveals complex effects of plant extracts on ALP and GST activity in *Nosema*-infected bees, while the other extracts might initially stimulate detoxification (GST).

*Nosema ceranae* infection in bees has been shown to induce energetic stress and malnutrition, leading to changes in gene expression, and enzyme activity^108,109^. From the toxicity assessment of the plant extracts to the uninfected healthy honeybees, selective targetability to the *Nosema* infecting the bees, biochemical and physiological properties of the bees, the methanolic extract of *A. nilotica* had the most affordable anti-nosemosis activity and less toxicity to the honeybees. So, the methanolic extract of *A. nilotica* has been further chemically analyzed to elucidate the most active constituents.

The GC–MS analysis of the methanolic extract of *A. nilotica* reveals the presence of several bioactive compounds namely myristic acid, 2,4,6-trihydroxybenzoic acid, 1,2-dihydroxybenzene, 2-butyloctanoic acid, phytol, butein, oleic acid, and rhamnetin. Myristic acid was found to be the major component, recognized with their high antimicrobial activities and potential benefits for bee health. The potential anti-Nosemal activity of myristic fatty acid could be by the selective disrupting of the cell membrane of *Nosema* spores, as consistent with previous antimicrobial studies of *Acacia nilotica*^38,39,41,76,110^**.** Myristic acid demonstrated a significant antimicrobial activity against bacteria and fungi^111–113^. Similarly, butyric and butyloctanoic acids have been found to have antimicrobial activity against *Salmonella typhimurium* and *Clostridium perfringens*^114^. Phenols in *Meliaceae* species were recognized with its insecticidal activities against *Spodoptera frugiperda* and *Plutella xylostella*^115,116^.

The docking analysis of the most frequent components of *A. nilotica* extracts “2,4,6-trihydroxybenzoic acid, 1,2-dihydroxybenzene, myristic acid, and linoleic acid”, were conducted to illustrate their binding affinity to the ATP binding protein, methionine aminopeptidase 2 and polar tube protein 2 as target enzymes of *Nosema* species. The binding affinity of 2,4,6-trihydroxybenzoic acid, 1,2-dihydroxybenzene, myristic acid and linoleic acid for ATP binding proteins of *Nosema* were − 5.2, − 4.5, − 4.4 and − 4.3 kcal/mol, respectively. The binding affinity of 2,4,6-trihydroxy-benzoic acid was lower than fumagilin by about 30%, ensuring the partial efficiency of the tested compounds. The binding affinity of 2,4,6-trihydroxybenzoic, 1,2-dihydroxybenzene, and myristic acid with methionine aminopeptidase of *N. apis* was − 5.1 kcal/mol, while linoleic acid had − 5.6 kcal/mol. The binding affinities of 2,4,6-trihydroxy-benzoic acid, linoleic acid, myristic acid and 1,2-dihydroxybenzene were − 6.8, − 6.9, − 6.3, and − 5.4 kcal/mol, respectively, compared to fumagillin (-8.8 kcal/mol) to the polar tube protein 2 of *N. ceranae*.

## Conclusion

*Nosema (Vairimorpha)* species were isolated from the honeybees, and the methanolic extracts of *Acacia nilotica* had the most selective activity to attack *Nosema* than honeybees. The titer of *N. apis* and *N. ceranae* was noticeably decreased by about 80- 90%, in response to *A. nilotica*, compared to the control. From the GC–MS analysis, the most frequent compounds of *A. nilotica* were 2,4,6-trihydroxybenzoic acid, 1,2-dihydroxybenzene, myristic acid, and linoleic acid. The molecular docking analysis of these compounds was conducted to illustrate their binding affinity to the ATP binding protein, methionine aminopeptidase 2 and polar tube protein 2 as target enzymes of *Nosema* species. From the molecular interactions, the compound 2,4,6-trihydroxybenzoic acid had the highest binding affinity to ATP binding proteins, methionine aminopeptidase and polar tube protein 2 of *N. ceranae,* compared to fumagillin as authentic anti-nosemal compound. From the experimental and molecular docking analysis, the methanolic extract of *Acacia nilotica* could be an alternative promising approach for the control of nosemosis of honeybees.

## Data Availability

All the data are available in the manuscript.
